# Optical Fibre-Based Sensors—An Assessment of Current Innovations

**DOI:** 10.3390/bios13090835

**Published:** 2023-08-22

**Authors:** Svetlana N. Khonina, Nikolay L. Kazanskiy, Muhammad A. Butt

**Affiliations:** 1 Samara National Research University, 443086 Samara, Russia; 2 IPSI RAS-Branch of the FSRC “Crystallography and Photonics” RAS, 443001 Samara, Russia

**Keywords:** optical fibre, biosensing, photonic sensor, plastic optical fibre, photonic crystal fibre, D-shaped fibre, U-shaped fibre, S-shaped fibre, fibre Bragg grating

## Abstract

Optical fibre sensors are an essential subset of optical fibre technology, designed specifically for sensing and measuring several physical parameters. These sensors offer unique advantages over traditional sensors, making them gradually more valuable in a wide range of applications. They can detect extremely small variations in the physical parameters they are designed to measure, such as analytes in the case of biosensing. This high sensitivity allows them to detect subtle variations in temperature, pressure, strain, the refractive index of analytes, vibration, and other environmental factors with exceptional accuracy. Moreover, these sensors enable remote sensing capabilities. Since light signals are used to carry information, the sensing elements can be placed at distant or inaccessible sites and still communicate the data back to the central monitoring system without signal degradation. In recent times, different attractive configurations and approaches have been proposed to enhance the sensitivity of the optical fibre-based sensor and are briefly explained in this review. However, we believe that the choice of optical fibre sensor configuration should be designated based on the specific application. As these sensors continue to evolve and improve, they will play an increasingly vital role in critical monitoring and control applications across various industries.

## 1. Introduction

Over the past 30 years, there has been a significant advancement in research on chemical and biological sensors [1]. For environmental, clinical, or security purposes, much of this research is now being conducted on the development of systems that can detect many analytes in a single sample [2,3,4]. The ability of optical sensors to probe surface coatings utilizing a variety of optical phenomena while attaining low noise and high sensitivity gives them considerable promise in this area [5]. They also allow for in situ sensing and real-time measurements and offer benefits in terms of speed. Miniaturization, remote sensing, and multi-analyte sensing are supplementary applications for optical sensors [5,6,7,8]. In comparison to electrical transduction systems, optical sensor systems have a reduced likelihood of producing an explosion in a hazardous environment and are thus freer from electromagnetic (EM) interference [9]. As a result, concerning other sensing systems, optical biosensors have a few benefits over laboratory-based systems [10,11,12].

Fibre optics makes use of the total internal reflection (TIR) concept, which allows for a correlation between the light intensity assessed at the detector and the initial target concentration [5]. For interaction with the target analyte, bio-receptors, for example, oligonucleotides, antibodies, and enzymes, can be immobilized on the core surface of the fibre [13]. Following the establishment of a reference standard curve, this interaction will affect the sensitive layer’s characteristics and be associated with the analyte concentration. Fibre optic biosensors have the advantages of high sensitivity, resistance, rapid detection, high sensitivity, and real-time monitoring and are unaffected by EM interference [5]. These qualities help fibre optic biosensors work well; because they can concurrently and discretely direct light of several wavelengths, they may be utilized for several analyte detections utilizing various DNA probes [14,15].

Optical fibre sensing techniques take advantage of the interaction between light and the fibre’s properties to enable accurate and reliable measurements. Some of the optical fibre sensors that work on different mechanisms, include fibre Bragg grating sensors (FBGs), Fabry–Perot sensors, interferometric fibre sensors, Raman scattering-based sensors, Brillouin scattering-based sensors, optical time-domain reflectometry (OTDR), polarimetric sensors, evanescent wave sensors, and optical frequency domain reflectometry (OFDR).

FBGs are periodic variations in the refractive index of an optical fibre’s core. When exposed to changes in strain or temperature, the Bragg wavelength (the wavelength of light that is reflected) shifts [16]. This shift is used to measure strain, temperature, pressure, and other parameters (details can be found in Section 4).

Fabry–Perot interferometers use a partially reflective surface (usually a thin film) to create an interference pattern between incoming and reflected light. Changes in the gap between the reflecting surfaces due to strain, pressure, or temperature variations lead to changes in the interference pattern, which can be measured to determine the parameter being sensed [17].

Interferometric fibre sensors use interference patterns in the light that travels through the optical fibre. This can include Mach–Zehnder interferometers and Michelson interferometers, where changes in the fibre’s properties cause phase shifts in the interfering light waves [18].

Raman scattering occurs when light interacts with molecules in the fibre, leading to a shift in wavelength proportional to temperature or strain. By analyzing this shift, temperature or strain can be measured along the fibre length. Brillouin scattering involves the interaction between light and acoustic waves in the fibre. The frequency shift of the scattered light provides information about temperature and strain changes along the fibre [19].

Optical time-domain reflectometry (OTDR) is a technique that uses backscattered light to measure the properties of an optical fibre. It is commonly used for distributed sensing of events like fibre breaks and bends [20].

Polarimetric sensors measure changes in the polarization state of light travelling through an optical fibre [21]. Changes in temperature, strain, or other parameters can alter the polarization, allowing these quantities to be determined.

Evanescent wave sensors exploit a small fraction of light that extends beyond the core of an optical fibre. By allowing this light to interact with the surrounding environment, changes in refractive index due to external factors can be measured [22].

Optical frequency domain reflectometry (OFDR) measures the frequency response of an optical fibre along its length. This technique provides high-resolution measurements of various parameters, including strain and temperature [23].

These techniques offer different levels of sensitivity, spatial resolution, and suitability for various applications. Fibre sensing technology continues to evolve, leading to innovations in sensor design and performance.

Optical fibre biosensors have undergone significant advances in recent years, offering numerous benefits for various applications [24,25]. Researchers have made substantial progress in improving the sensitivity and detection limits of optical fibre biosensors. This has been achieved through the development of novel sensing mechanisms, such as surface plasmon resonance (SPR) and localized surface plasmon resonance (LSPR), which enable extremely sensitive recognition of analytes at minimal concentrations [26,27]. Functionalization techniques have evolved to enhance the selectivity and specificity of optical fibre biosensors [28]. Various methods, such as immobilizing specific biomolecules on the fibre surface or incorporating functional nanomaterials, enable targeted detection of analytes, including proteins, DNA, and pathogens [10].

Miniaturization is a key area of advancement in optical fibre-based sensors. Researchers have been able to fabricate miniature optical fibres with diameters as small as a few micrometres, allowing for integration into microfluidic devices and other compact systems [29]. Miniaturization enhances portability, facilitates point-of-care testing, and enables in vivo sensing [30]. Progress in optical fibre biosensors has enabled the development of multiplexed sensing platforms [31,32]. By incorporating multiple sensing elements or using different sensing principles within a single fibre, it is now possible to detect multiple analytes simultaneously. This has applications in medical diagnostics, environmental monitoring, and food safety [33]. Improving the stability and biocompatibility of optical fibre biosensors is crucial for their integration into biological systems. Researchers have established coatings and surface modifications that boost sensor stability, reduce biofouling, and minimize interactions with biological samples, ensuring reliable and long-term operation [34,35,36].

Optical fibre biosensors now offer real-time monitoring and imaging competences. By integrating imaging fibres or utilizing optical coherence tomography (OCT) techniques, researchers can envisage and track biological processes and dynamic fluctuations in analyte concentrations in real time [37]. Optical fibre-based biosensors can be integrated with lab-on-a-chip systems, allowing for the creation of compact, portable, and highly efficient sensing platforms. This integration enables sample handling, processing, and analysis in a single device, simplifying the overall sensing process. These sensors find applications in various fields, including biomedicine and environmental monitoring. They are used for detecting biomarkers in bodily fluids, monitoring drug levels in patients, assessing water quality, and detecting pollutants in the environment [11].

These advances in optical fibre-based sensors hold great potential for enhancing diagnostic capabilities, enabling real-time monitoring, and improving the accuracy and efficiency of biological and environmental sensing applications. Two fundamental strategies can be taken into consideration depending on how the fibre waveguide’s structure is being optimized. The first involves varying the properties of the inner guiding wave using a particular kind of optical fibre as a probe. The second method involves applying additional handling and processing to the optical fibre’s structure to alter the guiding wave shape. Since optical fibre is so widely used, multi-mode fibre (MMF) [38] and single-mode fibre (SMF) [39] have been combined into a single fibre structure to significantly lower the cost of sensing devices. Conversely, numerous special fibre structures with fabricating properties, a controllable mode, and ease of integration, including polymer optical fibre (POF) [40], multi-core fibre (MCF) [41], photonic crystal fibre (PCF) [42], and hollow core fibre (HCF) [43,44,45], among others, have been suggested and manufactured for a variety of plasmon detection purposes.

It is challenging for evanescence waves to reach the cladding surface because incident light travels in the fibre’s core, which is enclosed inside the cladding. Due to this, it is commonly required to modify the fibre optic structure’s shape using a unique processing technique to disrupt the original optical transmission mode. In recent years, a growing number of fibre architectures, including the fibre grating structure [16,46], tapered optical fibre (TOF) [47], D-shaped fibre [48], and U-shaped fibre [49,50], have entered the scene. Appropriately specialized optical fibre processing machinery is required to realize these various optical fibre architectures.

Optical fibre-based plasmonic sensors offer several advantages over traditional fibre sensors, such as high sensitivity, miniaturization, remote sensing capabilities, and the ability to perform real-time monitoring [1]. They find applications in several fields, including environmental monitoring, biomedical diagnostics, food safety, and chemical analysis. It is important to note that the development and implementation of optical fibre-based plasmonic sensors involves complex engineering and fabrication methods. Additionally, the choice of plasmonic materials, sensing elements, and detection methods can vary depending on the specific application requirements. The sensitivity and specificity of plasmonic sensors for low-dose analyte detection must be better due to the growing demand for sensor applications, principally in life science, clinical diagnostics, medicine, and food safety. SPR/LSPR technology in the detection system has thus been focused on increasing sensitivity and detection range. A light source, fibre probe, and spectrometer are the typical components of an SPR/LSPR-based fibre analyte system. The evanescence waves generated by the light flow stimulate SPs at the interface between the metal layer and the sensor layer. The greatest coupling effectiveness between the evanescent field and surface plasmon waves depends significantly on the light wavelength, probe geometry, fibre parameters, and metal layer properties. Considering the aforementioned problems, optimizing the fibre waveguide’s shape and the immobilization effects of the coating materials are the two most effective ways to improve the sensing performance of SPR/LSPR sensors [51,52,53].

The paper is organized in the following manner: Section 2 presents the market and implications for fibre optic-based sensors. The sensing mechanism in optical fibre involves exploiting variations in light intensity, phase, wavelength, or polarization caused by the external factors being measured. The working principle of fibre optic sensors is presented in Section 3. Later, Section 4 presents the recent advancements in the most widely used configurations of optical fibre, such as photonic crystal fibre (PCF), S-shaped fibre, D-shaped fibre, U-shaped fibre, plastic optical fibre (POF), fibre Bragg grating (FBG)-based sensors. The article finishes with concluding remarks presented in Section 5.

## 2. Optical Fibre-Based Sensor Market and Implications

Since the 1980s, the use of fibre-optic sensors has steadily expanded across a range of applications. The underlying sectors have seen considerable advancements in distributed fibre-optic sensors, which provide continuous and real-time measurements over the whole length of an optical fibre cable [54]. Distributed fibre-optic sensors have substantial value throughout a well’s life cycle and may monitor pipelines that carry hydrocarbons over long distances in the oil and gas industry [55]. Distributed fibre optic sensors have several advantages over traditional electromechanical-based sensors used in the environmental, mining, civil, and geo-energy fields, particularly for harsh and difficult-to-access conditions [54,56,57,58]. These advantages include light weight, small size, electromagnetic interference immunity, remote detection, resistance to high pressure and high temperature, multiplexing, and smart sensing. Due to these factors, distributed fibre optic sensors are an optimum solution for displaying temperature, micro-seismic, and minor deformation changes caused by geologic CO_2_ sequestration over vast distances.

**Figure 1:**
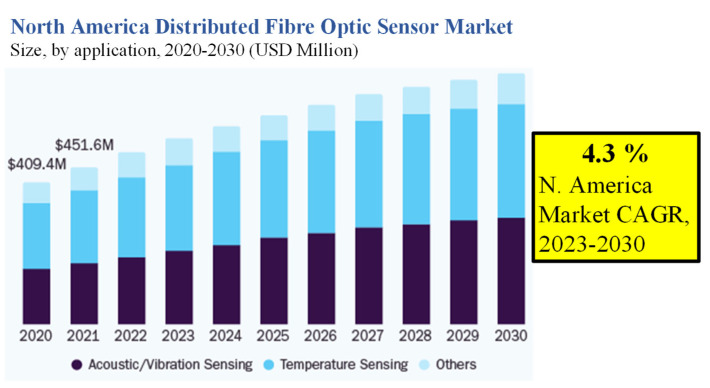
North America Distributed Fibre Optic Sensor Market [59].

The market for distributed fibre optic sensors was estimated at USD 1.44 billion in 2022, and from 2023 to 2030, it is anticipated to rise at a CAGR of 6.8% [59]. The market is seeing development prospects due to the increasing demand from businesses and companies to perform efficient sensing operations on their machine systems, as shown in Figure 1. Fibre optic sensing is now extensively used in a variety of commercial fields, including the automobile, aircraft, civil, energy, and other industries [60,61,62]. Rayleigh’s effect-based and Raman effect-based sensing are two more types of sensing technologies that have unique operating properties.

Distributed fibre optic sensors (DFOS) are very functional, which is driving more businesses to invest in technology and conduct R&D activities [63]. This leads to the creation of new items, giving businesses the chance to increase their industry share. Businesses want to improve productivity and control efficiency so that fibre optics technology will prevail over all alternatives. The high cost of DFOS product deployment and installation encourages businesses to create more dependable, competitively priced optic inspection devices.

By performing quality inspections of accuracy, range, and resolution, technologies like optical time domain reflectometry (OTDR) and optical frequency domain reflectometry (OFDR) now characterize fibre optics in the industry. Other notable changes include the use of cutting-edge technology like real-time thermal rating (RTTR) and intelligent distributed acoustic sensor (iDAS) technologies. The industry is seeing an increase in expenditures for R&D to produce revolutionary fibre cables and offer dependable connections at high speed and the most affordable pricing [64].

Optical fibre sensors are known for their high sensitivity. They can detect even small changes in physical variables such as temperature, pressure, strain, or refractive index [41]. This makes them valuable in various applications where precise measurements are required. Unlike traditional electronic sensors, optical fibre sensors are immune to EM interference. This property is particularly advantageous in environments with high electromagnetic noise, such as industrial settings or areas with strong radio frequencies. One of the key market drivers is the technology’s ability to provide top performance in difficult-end applications. Professionals can monitor and manage large-scale activities employed in pipeline construction, border security, and civil engineering thanks to optical fibres. Due to its high transmission capacity, the use of fibre optic cables provides businesses with better cost-saving potential than metal wires [65]. Companies are more likely to use the technology when it is deployed because of its high efficiency and fast transmission capabilities in remote and inaccessible locations.

In particular, the unique characteristics of optical fibre sensors make them indispensable in a wide range of industries, including telecommunications, aerospace, energy, manufacturing, environmental monitoring, and healthcare [66,67,68]. Their reliability, accuracy, and versatility continue to drive their importance in modern times [69].

Many companies are involved in the commercialization of optical fibre sensors. Some well-known players in this field include: (a) FISO Technologies, which offers fibre optic sensing solutions for medical, industrial, and energy applications [70]; (b) Luna Innovations, which provides fibre optic sensing solutions for aerospace, energy, and structural health monitoring [71]; (c) Micron Optics, which offers a range of fibre optic sensor products for various industries, including industrial monitoring and civil engineering [72]; (d) OSENSA Innovations, which specializes in fibre optic sensing solutions for the oil and gas, aerospace, and industrial sectors [73]; (e) Opsens, which provides fibre optic sensors for medical applications, including cardiovascular procedures [74]; (f) Neubrex, which focuses on distributed temperature and strain sensing technology [75]; and (g) Sensornet, which offers a variety of fibre optic sensors for industrial applications, particularly in the oil and gas industry [76].

## 3. Working Mechanism of Optical Fibre-Based Sensors

An optical fibre is a cylindrical waveguide mostly formed of silica (SiO_2_) that uses TIR to carry light down the fibre axis. A core (dielectric material) and cladding (another dielectric material with a lower refractive index) make up an optical fibre. The evanescent field, which penetrates the cladding when light is internally reflected, is a tiny component of light that may be created. The evanescent field at the fibre surface decays to a value that is almost negligible when the fibre cladding is significantly thicker than the fibre core. The EM field interacts with the surrounding medium when an optical fibre is tapered down to less than the core diameter, allowing light to pass through the cladding (rather than the core) and increasing the amplitude of the EM field in the tapered area [77,78,79].

**Figure 2:**
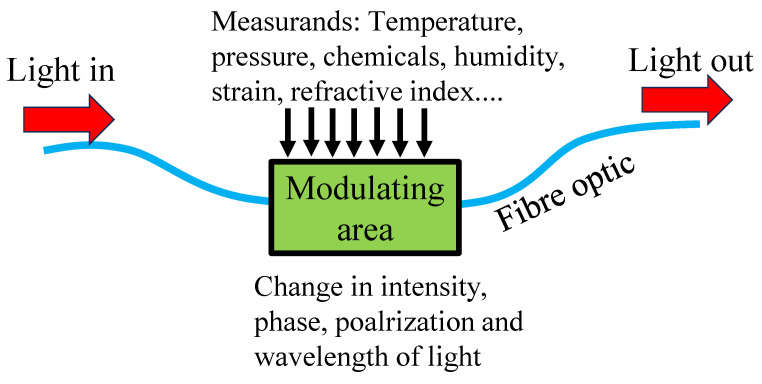
Working mechanism of fibre optic-based sensors.

The sensing mechanism in fibre optics involves exploiting changes in light intensity, phase, wavelength, or polarization initiated by the external factors being measured, as illustrated in Figure 2. Here are a few commonly used optical fibre sensing mechanisms:

(a) Intensity-based sensing: In this mechanism, the deviation in the intensity of light transmitted through an optical fibre is utilized to measure variations in the surrounding environment. It can be achieved by utilizing the transformation in light absorption, scattering, or reflection due to the target variable. Intensity-based sensors are straightforward and often used for applications such as temperature sensing or the detection of strain or pressure.
(b) Phase-based sensing: In phase-based sensing, the phase shift of the light wave travelling through the optical fibre is utilized to measure the target variable. The phase shift can be induced by changes in length, refractive index, or birefringence of the fibre. Phase-based sensors are commonly used for applications like vibration sensing, displacement measurement, or acoustic sensing.
(c) Wavelength-based sensing: This mechanism utilizes the changes in the wavelength of light propagating through the optical fibre to measure the target variable. It can be achieved by using fibre Bragg gratings or interferometric techniques. Wavelength-based sensors are widely employed for applications like strain sensing, temperature monitoring, or chemical sensing.
(d) Polarization-based sensing: Polarization-based sensing exploits changes in the polarization state of light travelling through the fibre to measure the target variable. These sensors often use fibre components such as polarizers, wave plates, or polarization-maintaining fibres. Polarization-based sensors are used in applications such as stress or strain sensing, current or magnetic field measurement, or biomedical sensing.

These sensing mechanisms can be combined or adapted to various types of optical fibre sensors to measure a wide range of physical quantities such as temperature, strain, pressure, humidity, chemical composition, and more [80,81,82]. The choice of sensing mechanism depends on the specific application requirements and the target variable being measured [83].

Optical fibre sensors are categorized in the literature considering many factors [84]. These sensors are usually categorized based on the operating theory or application as well as the position of the sensor within the fibre. When it comes to application, optical sensors can be divided into three categories based on the types of variables they are meant to measure: physical (such as strain and temperature), chemical (such as oil variables, pH, ammonia, detergents, and pesticides) [85], or bio-medical (such as oxygen, carbon dioxide, proteins, cells, and DNA) [84]. The optical sensor can be characterized as intrinsic or extrinsic depending on where it is located.

**Figure 3:**
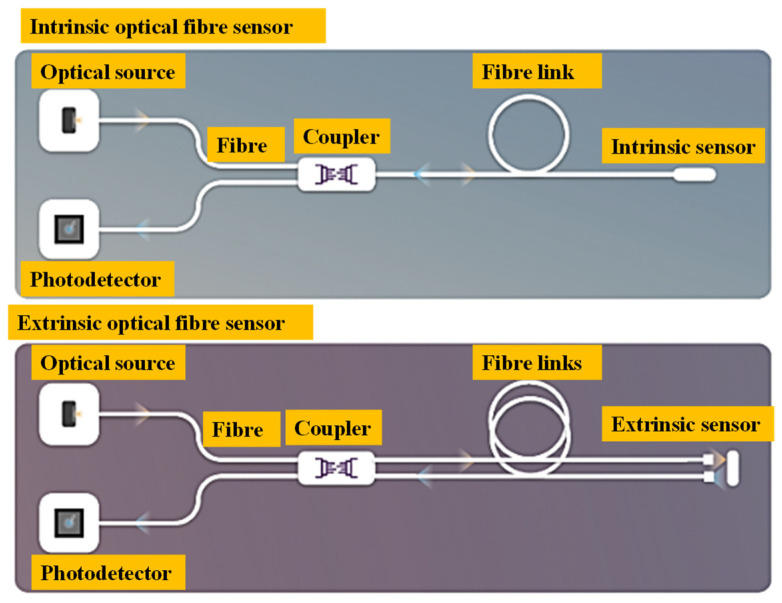
Types of optical fibre sensors based on the location of the sensor: intrinsic optical fibre sensor (**top**) and extrinsic optical fibre sensor (**bottom**) [86].

In an intrinsic sensor, the sensitive material (sensor head) and the channel for transmitting the optical signal containing the information measured are both directly made of an optical fibre, as shown in Figure 3 (top). They work by directly modulating the light that has been directed into the optical fibre; the light only exits the fibre at the detecting end. In this kind of sensor, physical disturbances alter the properties of the optical fibre, which alters the characteristics of the light it carries. As an alternative, the modulated light may be reflected or scattered back into the same fibre and then directed back to the detecting system. The simplest fibre sensors merely need a light source and a detector to alter the light’s intensity.

An extrinsic or hybrid optical fibre sensor directs light to and from the optical sensor head’s position (often based on a multimode fibre cable), as shown in Figure 3 (bottom). The sensor head, which is external to the optical fibre and is built from a tiny apparatus, is used to control the characteristics of light in response to alterations in the environment brought on by physical disturbances of interest. One end of the fibre transmits optical energy to the sensors’ heads, and the other end is modified and linked to the optical sensor.

The figure of merit (FOM) of an optical fibre sensor is a quantitative measure used to assess the sensor’s performance in terms of its sensitivity, precision, and overall effectiveness in measuring a specific parameter. Different types of optical fibre sensors may have different FOMs depending on the parameter being measured and the sensor’s design. The sensitivity of an optical fibre sensor refers to its ability to detect and measure small deviations in the quantity it is considered to sense. It is a measure of how responsive the sensor is to variations in the sensed variable. In other words, sensitivity quantifies the relationship between the change in the output signal of the sensor and the corresponding change in the sensed quantity. Sensitivity is typically articulated as a ratio or percentage, representing the change in the output signal per unit change in the sensed quantity [87].

The sensitivity of an optical fibre sensor can vary provisionally to various factors such as the design, construction, and operating principles of the sensor. Factors that can influence sensitivity include the type of sensing mechanism, the properties of the fibre (e.g., core size, numerical aperture), the detection method used, and environmental conditions. Higher sensitivity implies that the sensor can detect smaller changes in the sensed quantity [66]. It is an imperative variable to consider when selecting or designing an optical fibre sensor for a specific application. However, it is worth mentioning that sensitivity alone is not the sole determinant of a sensor’s performance. Other factors such as accuracy, resolution, dynamic range, response time, and stability also play crucial roles in evaluating the overall performance and suitability of an optical fibre sensor for a specific application [88,89]. Resolution is the smallest detectable change in the parameter that the sensor can reliably measure. It is related to the sensor’s noise level and determines the smallest incremental change in the parameter that can be distinguished from the sensor’s background noise [90].

The dynamic range of a sensor is the range of parameter values over which it can provide accurate measurements. A wide dynamic range allows the sensor to measure both small and large changes in the parameter without saturating or losing accuracy [91], whereas accuracy represents how close the sensor’s measurements are to the true value of the parameter being measured. It considers systematic errors and uncertainties introduced by the sensor’s design, calibration, and environment.

The response time of an optical fibre sensor is also important and refers to the time it takes for the sensor to detect and provide a meaningful output in response to a change in the parameter. Faster response times are often desirable, especially for dynamic or rapidly changing parameters [92].

Selectivity of the fibre sensor is vital, as it signifies the ability to specifically respond to a particular target analyte or parameter while minimizing or ignoring the effects of interfering substances or environmental factors [93].

Last but not least, the fibre sensor should be stable, which indicates the sensor’s ability to maintain its calibration and accuracy over time. Stable sensors retain their performance characteristics without significant drift.

## 4. Most Widely Employed Optical Fibre Configurations in Sensing Applications

**Figure 4:**
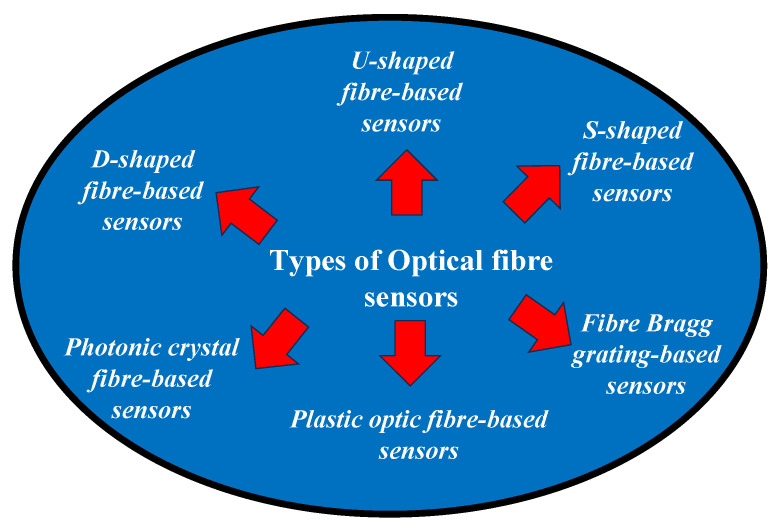
Types of optical fibre-based sensors reviewed in this paper.

In this section, six different kinds of optical fibre configurations are discussed for sensing applications, as shown in Figure 4. These specialized optical fibre configurations offer unique advantages for specific sensing scenarios. They enable efficient interaction with the target material or environment, allowing for accurate and localized measurements of various variables such as refractive index, proximity, or strain. The specific design and application of these sensors can vary depending on the desired sensing requirements and the targeted physical quantity to be measured.

### 4.1. Photonic Crystal Fibre (PCF)-Based Sensors

Photonic crystal fibre (PCF), also known as photonic bandgap fibre, is a type of optical fibre that uses a periodic arrangement of tiny air holes running along its length to confine and guide light [94]. Unlike traditional optical fibres that rely on TIR, PCF achieves light guidance through the phenomenon of photonic bandgap, which is like the forbidden energy bands found in electronic materials. The periodic arrangement of air holes in PCF creates a bandgap, which prevents the propagation of certain wavelengths of light. The guided modes are confined within the solid core region, allowing precise control over the fibre’s optical properties [95]. PCF offers excellent light confinement, enabling the guidance of light in a small solid core with a low refractive index. This confinement can result in enhanced light–matter interactions and improved nonlinear optical effects. By changing the size, shape, and distribution of the air holes, the optical properties of PCF can be tailored to meet specific application requirements. This flexibility allows for the design of fibres with exceptional dispersion characteristics, mode profiles, and nonlinear properties [96].

Like standard optical fibres, the PCF manufacturing process begins with the assembly of a preform. The preform is a solid glass rod with a specific arrangement of airholes and a desired refractive index profile. The preform can be made by employing various techniques, including stack-and-draw, drilling, or extrusion. In the stack-and-draw method, a series of hollow glass tubes, also known as capillaries, are stacked around a central glass rod to form the desired airhole pattern. The capillaries can have different sizes and shapes depending on the anticipated PCF structure. The assembled stack is then heated and drawn into a long fibre while maintaining the arrangement of airholes. In the drilling procedure, a solid glass rod is first manufactured. Then, a series of precisely controlled laser pulses or mechanical drilling is used to create the desired pattern of airholes directly into the glass rod. The extrusion technique involves the use of a specialized die that has a series of small holes arranged in the desired pattern. The glass material is heated and forced through the die to form the PCF structure with airholes. Once the preform is prepared, it is heated in a high-temperature furnace and drawn into a long, thin fibre while maintaining the arrangement of airholes. The drawing process involves carefully controlling the temperature, tension, and speed to realize the desired dimensions and optical properties. Like conventional fibres, a protective coating is applied to the PCF to provide mechanical strength and environmental protection. The coating is characteristically made of a polymer material and applied using various coating techniques such as UV curing or thermal curing. After manufacturing, the PCF undergoes extensive characterization tests to assess its optical properties, such as mode structure, dispersion, and loss. These tests help ensure that the PCF meets the required specifications for its intended applications.

Achieving accurate and consistent control over the microstructure of the PCF, including the shape, size, and arrangement of air holes, is critical for obtaining the desired optical properties. Even slight variations can result in changes to the fibre’s modal properties, dispersion characteristics, and sensitivity. Moreover, PCFs are typically made from special materials with tailored optical properties. These materials might be more challenging to work with compared to traditional optical fibre materials, requiring specific fabrication techniques and tools.

PCFs can support multiple modes of light propagation, which can complicate sensor design and operation. Achieving single-mode behavior or controlling the interaction between modes can be technically demanding. If the PCF is intended to be connected to other optical components, achieving proper end-face polishing and alignment while maintaining the intricate photonic crystal structure can be challenging. Tapering or splicing PCFs to other fibres or components can be tricky due to the varying air-hole patterns and structural changes along the fibre length.

PCF can also be designed to have a larger mode area compared to conventional fibres, reducing nonlinear effects such as optical nonlinearities and stimulated Brillouin scattering [97]. This feature makes PCF suitable for high-power laser delivery and nonlinear optics applications [98]. PCFs can be engineered to have a single mode across a broad wavelength range, ensuring excellent beam quality and reducing modal dispersion. This makes them suitable for applications that require high-quality and low-loss transmission, such as telecommunications and fibre lasers [42,99]. Nevertheless, PCF has found applications in various fields, including telecommunications, sensing, imaging, and nonlinear optics [100]. It has been used in areas such as supercontinuum generation, high-power fibre lasers, high-resolution microscopy, and fibre optic sensors [24,101].

**Figure 5:**
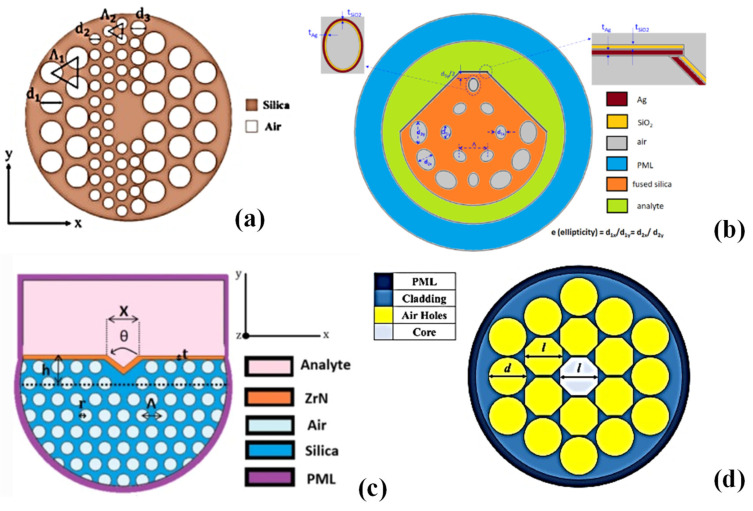
PhC-based sensor for (**a**) the detection of underwater temperature and salinity [102], (**b**) enhanced temperature sensing [104], (**c**) cancer detection [105], and (**d**) blood component sensing [106].

The simultaneous measurement of underwater salinity and temperature using an innovative, small fibre sensor with great sensitivity is suggested [102]. The structure is built on a highly birefringent, asymmetric, full-circular hole photonic crystal fibre (HB-A-FCH PCF), as shown in Figure 5a, which reaches a birefringence value of 2.83 × 10^−3^ at 2.2 µm in wavelength. Additionally, the temperature sensitivity of HB-A-FCH PCF is markedly enhanced by ethanol-filled air holes. To concurrently detect salinity and temperature using the spectrum responses of the x- and y-polarization state, a Michelson interferometer-based sensor setup is simulated with air holes acting as fluid microchannels. According to theoretical analysis, the sensor can detect salinity and temperature changes up to 5.1472 nm/% and −0.8987 nm/°C in the x-polarized fundamental mode, respectively, and up to 4.5386 nm/% and −0.8000 nm/°C in the y-polarized fundamental mode [102].

Plasmonics involves the interaction between light and surface plasmons, which are collective oscillations of electrons in a metal surface [7,81,103]. This interaction can result in enhanced light–matter interactions and sensitivity. In an optical fibre-based plasmonic sensor, the plasmonic sensing element is integrated with an optical fibre. The plasmonic element typically consists of metallic nanoparticles, nanowires, or thin films deposited on the fibre’s surface or within its core [82]. These plasmonic structures interact with the evanescent field of light propagating through the fibre, allowing the detection of analytes or changes in the surrounding environment [83]. Another highly sensitive temperature sensor is proposed based on PCF utilizing a flat metal-coated trapezoidal surface, as shown in Figure 5b [104]. A polished trapezoidal surface and two layers of elliptical air holes make up the PCF, which enables temperature detection. A thin SiO_2_ film serves as an oxidation-resistant coating, and a thin metal (Ag) layer is applied to the reflecting surface using an external sensing technique. The interface for energy conversion from the core-guided mode to the SPP mode is the top elliptical air hole. The suggested SPR-PCF temperature sensor may reach a maximum temperature sensitivity and resolution of up to 5.2 nm/°C and 0.01923 °C, respectively, within a temperature range of 10 to 60 °C, according to simulations conducted using the finite element approach.

An SPR-based PCF biosensor with a unique design is presented and examined for cancer cell detection [105]. The numerical analysis of the proposed biosensor makes use of the full vectorial finite element method (FVFEM). The disclosed PCF features a V-shaped surface that is covered in ZrN to act as a plasmonic material, as shown in Figure 5c. The examined analyte determines if there is a link between the surface plasmon mode (SPM) and the core-guided mode. The recommended V-shaped structure, which raises sensor sensitivity, improves such a connection. To obtain high sensor sensitivity, geometrical characteristics are optimized. The suggested biosensor can detect breast, basal, and cervical cancer cells with an optical sensitivity of 6214.28 nm/RIU, 3800 nm/RIU, and 5008.33 nm/RIU for TM-polarized mode, and 6000 nm/RIU, 4400 nm/RIU, and 5333.3 nm/RIU for TE-polarized mode, respectively. The described optical sensor might open the door to a quick, low-cost, high-sensitivity method of cancer diagnosis in place of surgical and chemical methods [105].

The identification of blood components, such as red blood cells, haemoglobin, white blood cells, plasma, and water, has been fully examined using a PCF-based sensor [106]. COMSOL Multiphysics was used to conduct a numerical study to assess the sensor’s sensing and propagation characteristics. An octagonal core and two layers of cladding with circular and octagonal air holes make up the suggested sensor design, as shown in Figure 5d. The thorough simulation results show that the suggested sensor achieves high relative sensitivity of 99.89%, 99.13%, 97.95%, 97.77%, and 96.68% for red blood cells, haemoglobin, white blood cells, plasma, and water, respectively, at the ideal wavelength of 7.0 µm. The design also exhibits good beam divergence, spot size, confinement loss, propagation constant, and V-parameter. The suggested PCF-based sensor hence offers considerable potential for optical communications as well as medical sensing applications. It is a useful instrument for a wide range of prospective applications in the biological and telecommunications areas because of its cutting-edge design and extremely sensitive capabilities.

### 4.2. D-Shaped Fibre-Based Sensors

D-shaped fibre refers to an optical fibre with a non-circular cross-section that resembles the letter “D” in shape. Unlike traditional round fibres, D-shaped fibres have one flat side, which presents unique characteristics and enables specific applications. D-shaped fibres have a flat side, typically achieved by flattening a round fibre or by fabricating a fibre with a non-circular core. The flat side can be oriented in a specific direction, allowing for controlled light coupling and interaction with other components. The flat side of a D-shaped fibre facilitates efficient coupling with other optical components, such as laser diodes, photodiodes, or waveguides [107]. The shape allows for precise alignment and positioning, enabling easy integration into various devices and systems.

D-shaped fibres are often used as a fibre-to-device interface, providing a convenient and stable connection between the fibre and the target device. The flat side can be directly mounted or bonded onto a substrate, ensuring reliable alignment and minimizing alignment losses. D-shaped fibres are usually employed for sensing purposes [108]. The flat side can be coated with sensing materials or placed close to the sensing element, enhancing the interaction between the guided light and the external environment [109]. This configuration enables applications such as evanescent wave sensing, refractive index sensing, and SPR sensing [110,111,112]. D-shaped fibres are also used in the fabrication of fibre Bragg gratings (FBGs). FBGs are periodic variations in the refractive index of the fibre core, acting as narrowband reflectors. The D shape allows for precise alignment of the grating structure, enabling efficient coupling of light into the grating region and enhancing the grating’s performance. D-shaped fibres are suitable for compact and integrated optical devices and systems. Their unique geometry allows for tight packaging and integration with other components in applications such as fibre optic switches, microfluidics, and optofluidics.

To detect variations in the refractive index of liquid analytes, an SPR sensor based on a D-shaped PCF is presented in [113]. The numerical simulation was performed using the finite element method (FEM). The performance of the SPR-PCF sensor with zinc oxide (ZnO) and graphene layer was estimated using silver as the plasmonic metal. The design of the sensor eliminates the need to fill the hole with material and to coat the hole wall by merely coating the polished surface, simplifying sensor manufacture. The influences of structural factors on sensor performance, including lattice spacing, graphene layer thickness, silver layer thickness, ZnO layer thickness, and manufacturing tolerance of blowhole diameter, were numerically simulated. The numerical findings demonstrated that the ZnO thickness range between 10 and 25 nm is where the SPR-PCF sensor’s sensitivity is at its greatest. The maximal sensitivity and corresponding resolution for a liquid analyte in the refractive index range of 1.37–1.41 are 6000 nm/RIU and 1.667 × 10^−5^, respectively. The sensor also has great structural tolerance and strong stability, with a blowhole diameter tolerance of 5%. This research has broad applications in the monitoring of water contamination, the detection of biochemical analytes, monitoring the quality of food, and medical diagnostics [113].

**Figure 6:**
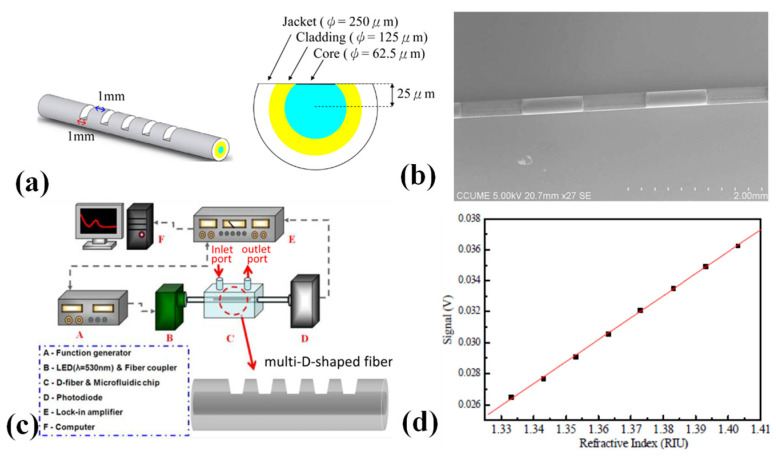
(**a**) Schematic of a multi- D-shaped optical fibre [114], (**b**) SEM image of the multi- D-shaped optical fibre [114], (**c**) experimental setup for refractive index measurement [114], (**d**) sensor response versus sucrose solution [114].

Refractive index measurements may be performed using a new class of multi-D-shaped optical fibre (Figure 6a) that is provided in [114]. By using femtosecond laser pulses to create several D sections in a multimode optical fibre at certain localized locations, the multi-D-shaped optical fibre was created. A SEM image of the multi-D-shaped fibre is shown in Figure 6b. Three to seven D-shaped zones may have been manufactured in all. A sensor volume of 100 µm in depth, 250 µm in breadth, and 1 mm in length was covered by each D-shaped zone. The core surface’s mean roughness, as determined by the AFM scans, was 231.7 nm, which is quite smooth. The fibres have enough mechanical strength, according to the results of the tensile test, to withstand damage from additional processing. It was discovered that a multi-D-shaped optical fibre may be used as a very sensitive refractive index sensor to track changes in the environment. The experimental setup for refractive index sensing measurements using a multi-D-shaped optical fibre sensor is shown in Figure 6c. A function generator, an LED light source, a sensing multi-D-shaped fibre, a microfluidic chip, a photodiode, a lock-in amplifier, and a computer for data collection made up of the fibre-optic sensing system used to assess the transmission power of the sensor. The sensor response versus different concentrations of sucrose is shown in Figure 6d. The outcomes for various sucrose solution concentrations demonstrated that a resolution of 1.27 × 10^−3^–3.13 × 10^−4^ RIU was attained for refractive indices in the range of 1.333 to 1.403, indicating that the multi-D-shaped fibres are appealing for chemical, biological, and biochemical sensing with aqueous solutions [114].

D-shaped fibres are often achieved through specific manufacturing processes, and they can present some challenges in their fabrication. The process of flattening the fibre needs to be precisely controlled to attain reliable results. The pressure, temperature, and duration of the flattening process can all impact the final shape and characteristics of the fibre. Creating a consistent and precisely shaped D profile requires tight manufacturing tolerances. Any deviations from the desired shape can affect the fibre’s performance, coupling efficiency, and overall functionality. The choice of materials for both the fibre core and cladding is critical. Materials that can be easily flattened without distressing their optical properties must be chosen to achieve the desired functionality. The core and cladding layers in an optical fibre must maintain their optical properties even after the fibre is flattened. Achieving this control can be challenging, as the flattening process may alter the refractive index profile of the fibre and lead to unwanted changes in optical characteristics. Guaranteeing uniformity in the flattened portion of the fibre is crucial for consistent performance. Variations in thickness or shape can lead to variations in light coupling efficiency, affecting the sensor’s accuracy or the fibre’s transmission properties.

The surface quality of the flattened portion is imperative for effective interaction with external elements, such as coupling optics or sensing materials. Uneven or rough surfaces can cause light scattering, which can impact signal transmission or sensing capabilities. If the D-shaped fibre is intended to be connected to other optical components, such as connectors or couplers, achieving proper end-face polishing while maintaining the desired shape can be challenging.

Flattened fibres might be more susceptible to mechanical stress or bending due to their altered geometry. Ensuring the mechanical integrity and long-term reliability of the fibre can be a challenge. Reproducing the desired D shape consistently across multiple fibres is essential for mass production and commercial viability. Variability between fibres can lead to inconsistent performance and difficulty in quality control. If the D-shaped fibre is intended for use as a sensor, integrating sensing elements or coatings onto the flattened portion while maintaining their functionality can be technically demanding.

### 4.3. S-Shaped Optical Fibre-Based Sensors

Optical fibres are typically designed to be as straight as possible to minimize signal loss and distortion. However, there are certain applications where intentionally bending or shaping the fibre into specific configurations can be beneficial. The S-shaped fibre structure is distinctive in that it bends during the stimulated high-order mode’s taper transition, which was initially suggested by Yang et al. for usage as a small MZI [115]. The structure’s parameters, such as the axial offset length, waist diameter, bending degree, and other factors, have an impact on the fibre structure’s transmission properties. The axial offset and tensile length of the fibre are mostly affected by the characteristics. As a result, extensive study may be performed to determine how varied stretching lengths and axial offset lengths affect the sensor’s transmission properties.

The effectiveness of S-shaped fibre sensors relies on the coupling of light between different modes of fibre due to bending-induced effects. Achieving consistent and repeatable mode coupling across different sensors and under various conditions can be challenging. Creating precise and reproducible S-shaped bends in the optical fibre requires careful control of the bending radius and curvature. Any inconsistencies can lead to variations in the mode coupling effect and sensor response.

S-shaped fibre sensors often require accurate calibration procedures to relate the measured changes in mode coupling to the physical parameter being sensed. Additionally, baseline correction may be needed to account for variations in the sensor’s response due to environmental conditions [116]. Moreover, S-shaped fibre sensors can exhibit cross-sensitivity to environmental factors beyond the targeted parameter. Variations in temperature, pressure, or other external influences can affect the sensor’s response, potentially leading to measurement errors.

S-shaped fibre sensors may not be well-suited for dynamic measurements or applications where the sensor experiences rapid or frequent changes in curvature. The time required for mode coupling and relaxation can limit their responsiveness, whereas S-shaped fibre sensors can be sensitive to temperature variations, which may require compensation techniques to isolate the targeted parameter from temperature-induced effects.

The S-shaped optical fibre configuration can be used in applications such as fibre optic sensing systems or distributed fibre optic sensing (DFOS) [117,118]. DFOS systems rely on the interaction between light and the optical fibre to detect changes in temperature, strain, or other physical variables along the length of the fibre. Shaping the fibre into an S curve can boost the sensitivity of the system to changes in the environment. The bending of the fibre introduces mechanical stress, which can affect the light propagation characteristics. This stress-induced alteration can be utilized to detect and measure changes in the external conditions that affect the fibre. The S-shaped fibre design allows for increased interaction between the light and the environment, enabling more precise measurements in sensing applications [119]. The bending of the fibre can create areas of increased strain or temperature sensitivity, which can be exploited for various sensing purposes. It is worth noting that the exact design and fabrication of S-shaped optical fibres can vary depending on the specific application requirements. Different techniques, such as fusion splicing or specialized fibre fabrication processes, may be used to create the desired shape while maintaining the optical and mechanical properties of the fibre. Overall, S-shaped optical fibres provide a means to boost the sensitivity and performance of fibre optic sensing systems by introducing controlled bending and shaping into the fibre structure [120].

To increase the sensitivity of an evanescent wave absorption-based fibre-optic biosensor, an S-shaped multimode fibre-optic probe has been used [121]. Human immunoglobulin (HIgG) and a goat anti-human immunoglobulin (GaHIgG) antigen-antibody combination were used for the immunosensing procedure. FITC was used as a reporter molecule in place of the enzyme during the direct ELISA format of the immunosensing. In another work [120], it was found that S-shaped fibre-optic probes had better evanescent wave absorption and refractive index sensitivity than U-shaped ones. The lowest measured concentration of the GaHIgG biomolecule was increased to 1.7 nM by the manufactured S-shaped geometry. The suggested immunosensor demonstrated very little non-specific binding on the sensor surface, increasing the detection precision.

**Figure 7:**
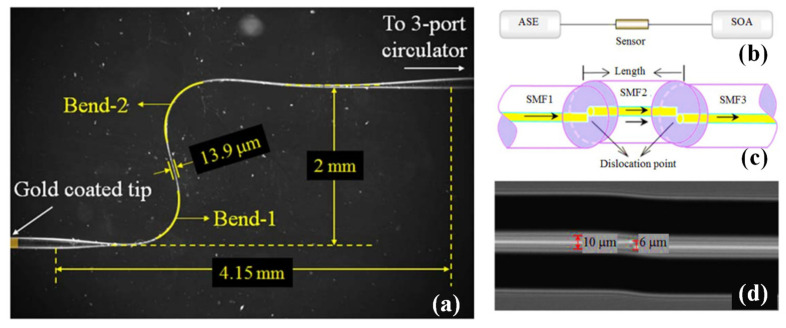
(**a**) Micrograph of STF [122], (**b**) schematic diagram of the proposed dislocation fibre MZI-based sensor measurement system [123], (**c**) schematic diagram of the dislocation fibre MZI-based sensor [123], and (**d**) microscope image of the point of dislocation fibre (6 μm) [123].

Based on S-shaped tapered fibre (STF), as shown in Figure 7a, a straightforward and incredibly sensitive optical microphone was presented [122]. A thin, round nitrile diaphragm is connected in the middle to the short, pigtailed end of the STF. The nitrile diaphragm is deformed by the applied acoustic signal, and because of the affixation, the STF structure is altered, changing the bending angles of the two STF bends. As a result, the output of the photodetector, which measures the intensity of the STF’s reflected light, changes in response to the supplied acoustic signal. By adjusting the diaphragm width and thickness as well as the shapes and sizes of the STF, it is simple to modify the suggested sensing setup’s various features. The suggested sensor achieves a sensitivity of 3.07 mV/Pa and a minimum detectable pressure of 36.48 mPa/Hz for an optimized setup. Up to 1300 Hz, the sensor exhibits linear behaviour, and its experimental first-order natural frequency is 1455 Hz [122].

A straightforward, small, and reliable temperature sensor based on an S-shaped displaced optical fibre, was developed [123]. As seen in Figure 7b, an optical spectrum analyser (OSA) (Ando AQ6317D2), an optical circulator, and an amplified spontaneous emission (ASE) source with a laser wavelength range of 1530 nm to 1560 nm were used to monitor the spectrum of the suggested sensor. Based on the theory and our experiment, it was possible to determine the ideal variables for the dislocation optical fibre’s two splice locations, including its length and dislocation amount. At the misalignment-spliced joint, SMF1’s light transmission is split in two, as shown in Figure 7c [123]. One portion of the light is connected to the core mode of SMF2, while the other portion is coupled to the cladding modes of SMF2. The lead-in SMF3’s core will be linked with the two components of the light. It was possible to create an MZI-based sensor in one fibre using the two portions of light of SMF2. The microscope image of the point of dislocation fibre is shown in Figure 7d [123]. The environment’s temperature may be determined based on the correlation between the temperature and the peak wavelength shift. The S-shaped micro-bending component may release stress with the temperature change and lessen the impact of tension on the temperature measurement; thus, we formed this fibre into an S shape between the two dislocation sites [123]. This arrangement may be able to address the issue of sensor distortion brought on by temperature and stress cross-responses. The S-shaped dislocation fibre had the benefits of steady dependability and strong linearity when we evaluated it beside a dislocation fibre sensor without an S shape in the same setting and under the same conditions.

### 4.4. U-Shaped Optical Fibre-Based Sensors

Like S-shaped fibres, U-shaped fibres are intentionally designed to have a specific curvature for various applications. U-shaped optical fibres can be utilized in a range of applications, including sensing, telecommunications, and biomedical fields. U-shaped fibres can be employed in sensing applications to detect changes in temperature, pressure, strain, or other environmental variables [124,125,126]. The bending of the fibre allows for increased interaction with the surrounding medium, enhancing the sensitivity of the sensor. For instance, U-shaped fibre sensors can be utilized for measuring the refractive index of liquids or detecting biochemical changes in biological samples.

**Figure 8:**
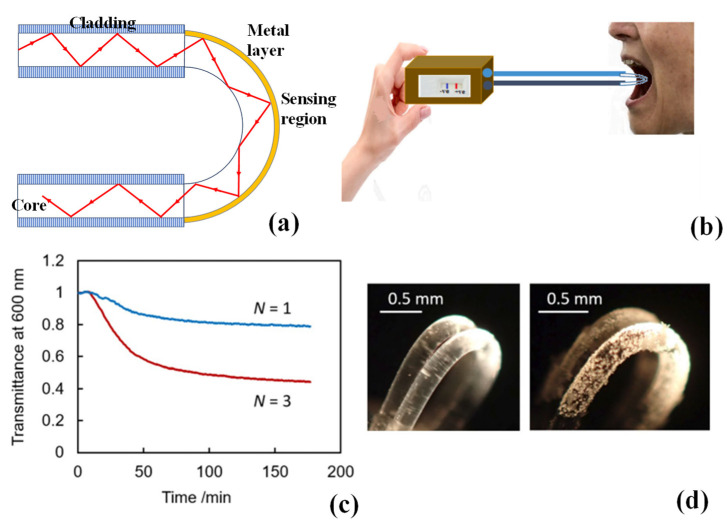
(**a**) Schematic illustration of U-shaped fibre. (**b**) Schematic of rapid detection via saliva [129]. (**c**) Transmittance responses of the one- and three-bend U-shaped POF sensors to CaCO_3_ scale deposition in hot spring water sampled at Matsushiro, Japan [130]. (**d**) Photographs of the surface of the three-bend sensor before (**left**) and after (**right**) CaCO_3_ scale deposition in the hot spring water sample [130].

The U-shaped optical fibre sensor implements the sensing concept using the two sensing modes. The TIR condition of the fibre core is no longer met if just the coating layer is removed, and the fibre is bent into a U shape by heating or fixing to the mould. When light exits the fibre core and enters the cladding layer, some of it is refracted to the outside, and some of it is reflected at the cladding–external environment interface, creating evanescent waves that may have an impact on the metal film coated on the fibre, as shown in Figure 8a. A type of U-shaped fibre sensor with a simpler operating principle may also be created by removing the fibre’s cladding before bending. It may be inferred that surface plasmon waves will be directly stimulated by the evanescent field produced by TIR in the fibre core. The original fibre core transmission mode is then altered by the U-shaped construction, and the effective evanescent absorption coefficient rises as the radius of curvature decreases [127].

The fabrication of U-shaped fibre sensors can be more intricate compared to simple straight-fibre sensors. Creating the U shape in the fibre requires careful handling and precise bending, which can lead to challenges in maintaining consistent sensor performance. U-shaped fibre sensors, like S-shaped optical fibre sensors, can exhibit cross-sensitivity between temperature and strain measurements. This cross-sensitivity can complicate accurate parameter measurement, especially in dynamic conditions. Moreover, U-shaped fibre sensors might not have standardized designs or calibration procedures, which can make it challenging to ensure consistent and comparable measurements across different setups.

The development of an optical fibre sensor with a U shape for temperature readings is described in [128]. The sensor was integrated into a U-shaped optical fibre sensor using flame heating and is based on single-mode fibres. To decrease the sensor layer’s susceptibility to humidity, this study coated the sensor layer with PVA, a polymer, using electrospinning. The sensor can monitor temperature changes between 30 °C and 100 °C. The goals of this study were to use COMSOL to model the wavelength signals produced at various electrospinning durations and to analyse the sensitivity variation of the sensor with varying sensor layer thicknesses resulting from those durations. According to the findings, the sensor’s linearity, maximum wavelength sensitivity, and transmission loss sensitivity were 25 dBm/°C, 70 pm/°C, and 0.956, respectively. Longer electrospinning times produced thicker sensor layers and greater sensor sensitivity, with a 42% increase in the wavelength sensitivity of the sensor [128].

U-shaped fibres can be used as optical switches or modulators. By applying external forces, such as pressure or electrical signals, to the bent region of the fibre, the optical characteristics of the fibre can be modified. This enables control over the light transmission through the fibre, facilitating functions like switching or modulation. U-shaped fibres find applications in biomedical fields, particularly in endoscopy and minimally invasive procedures. The U shape allows for compact and flexible fibre bundles that can be inserted into the body, providing imaging or therapeutic capabilities.

In the fabrication of U-shaped fibres, different techniques may be employed, such as fibre fusion splicing, lithography, or specialized fibre shaping methods. The goal is to achieve the desired bending radius and curvature while maintaining the optical properties of the fibre. Overall, U-shaped optical fibres offer versatility in terms of their applications, providing enhanced sensing capabilities, optical switching functionality, and flexibility for biomedical procedures. The specific design and usage of U-shaped fibres depend on the desired application and the requirements of the system or device in which they are used.

A novel idea for a flexible sensor system intended for the early detection of oral cancer was put forth in [129]. The probe can provide quantitative and qualitative evaluations using saliva samples based on intensity modulation. It may also credibly confirm the patient’s mouth cancer’s stage. Due to the inclusion of biomarkers, this catheter-like probe exhibits an exceptional reaction time and high specificity, which frees the user from sample storage issues and other related obstacles. As shown in Figure 8b, the probe is placed within the infected patient’s mouth for saliva examination. The intensity modulation becomes active because of significant contact with saliva. If the acquired reaction exceeds the threshold, the person whose saliva was sampled is diagnosed with oral cancer. The portable device’s incorporated optical and electrical interconnects, which take the form of a functioning detector that serves as both a receiver and a transmitter, provide qualitative and quantitative display results [129].

Fibre optic sensors for scale deposition monitoring in geothermal brine and hot spring water must be secure, simple to make, and simply disposed of. These desirable characteristics have previously been improved in U-shaped sensors and plastic optical fibres (POFs) for various applications. A U-shaped POF sensor for CaCO_3_ scale deposition is described in [130]. By thermally bending the bulk POF without removing the cladding, the sensors were quickly made. The high refractive index of the CaCO_3_ deposit affects the fraction of total internal reflection between the water and POF surface at the bend. Using a white light source and a spectroscopic detector, the optical responses of the U-shaped sensor to CaCO_3_ production were studied in a solution of calcium chloride dihydrate and sodium hydrogen carbonate. The sensor responded when CaCO_3_ crystallized on its surface, and it was particularly sensitive at tiny bending radii. By adding more bends, the sensitivity was increased even further. The monitoring of CaCO_3_ scale deposition in hot spring water collected in Matsushiro, Japan, was the final application of the U-shaped POF sensor [130]. Figure 8c displays the transmittance variations observed in hot spring water by the one- and three-bend sensors. Both sensors were able to detect scale deposition once the temperature reached 50 °C, although the three-bend sensor had better sensitivity. Additionally, pictures taken before and after the experiment showed the scale deposition on the sensor surface and light leaking from the deposits (see Figure 8d). The findings supported the sensor’s ability to pick up scale deposits that include CaCO_3_ in hot spring water [130].

### 4.5. Plastic Optical Fibre (POF)-Based Sensors

Plastic optical fibre (POF) is a type of optical fibre that is made of plastic materials, typically polymethyl methacrylate (PMMA). Unlike traditional glass optical fibres, which are made of silica, plastic optical fibres utilize polymer materials for their core and cladding. POF is generally less expensive to manufacture than glass optical fibres [131]. The materials used in POF production are more affordable, which contributes to lower overall costs. Plastic fibres are more flexible and resistant to breakage compared to glass fibres [132]. They can withstand bending and stretching without significant loss of performance, making them more durable and easier to handle. POF has a larger core diameter (typically around 1 mm) compared to the smaller core diameter of glass fibres. This larger diameter makes it easier to terminate and install connectors, reducing the complexity and cost of installation. Like other optical fibres, POFs are not affected by electromagnetic interference (EMI) since they do not conduct electricity. This property makes POF suitable for applications in electrically noisy environments. Moreover, POFs are not as brittle as glass fibres and are considered safer in certain applications. They do not shatter into sharp fragments if they break, reducing the risk of injury [133].

However, it is important to note that POFs have limitations compared to glass fibres. They have higher signal attenuation (loss) over longer distances, limited bandwidth capabilities, and lower tolerance for high temperatures. As a result, POF is often used in short-range communication applications, such as home networking, automotive networks, and industrial control systems, where the advantages outweigh the limitations. Compared to traditional glass optical fibres, POFs generally have lower sensitivity to changes in parameters like temperature and strain. This can limit their accuracy and precision in some measurement applications.

However, POF-based sensors can be used in various applications, including sensing physical variables like temperature, pressure, strain, humidity, and chemical concentrations [134]. The working principle of a POF-based sensor involves measuring changes in the light transmitted through the fibre due to the interaction with the external environment [135]. There are different types of POF-based sensors, such as intensity-based sensors, interferometric sensors, and fluorescence-based sensors [136,137]. In an intensity-based sensor, the detected light intensity changes in response to the measured variable, while interferometric sensors use the interference of light waves for sensing. Fluorescence-based sensors utilize the emission and absorption of fluorescent materials to detect specific substances.

**Figure 9:**
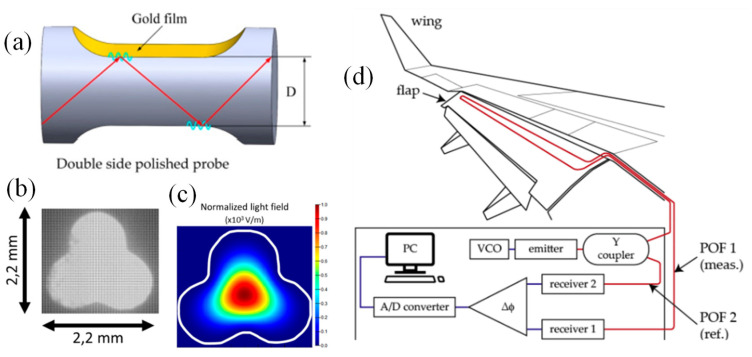
(**a**) Schematic of a double side polished fibre SPR probe [138]. (**b**) Optical microscope photograph of the cross-section of the three-lobe POF [139]. (**c**) Simulated light field distribution of the fundamental mode at 645 nm [139]. (**d**) Diagram of the POF elongation sensor, which measures how long an aeroplane flap has extended. The light signal is modulated using a voltage-controlled oscillator [140].

It is suggested to use a double-sided polished formation to create an improved POF-based SPR sensor [138]. The sensor is created by symmetrically polishing two sides of the POF along the fibre axis, after which a layer of Au film is placed on both sides of the polished area, as shown in Figure 9a. Using this configuration will result in a greater quantity of light reflections since the SPR may be activated on both polished sides with an Au film coating. The findings of the modelling and experiment demonstrate that the suggested sensor has an improved SPR effect. For a high measured refractive index, it is possible to enhance visibility and full width at half maximum (FWHM) of the spectrum. When the refractive index of the measured liquid is 1.42, the double-sided POF-based SPR sensor achieves a sensitivity of 4284.8 nm/RIU. The suggested SPR sensor is inexpensive to produce, has a wide measurement window and action area for the substances being monitored, and has potential use in the biochemical sensing and oil industries [138].

POF-based curvature sensors were initially developed primarily as intensity sensors. For instance, when a bend is applied, the fibre’s core is exposed, abrading a portion of the cladding to amplify the irradiation light. These sensors were used to track the curvature of human joints and the spine, to check the health of structures, or as breathing sensors. Nano displacement was also detected utilizing a double-core fibre and an interferometric method. A three-lobe PMMA fibre core without cladding was used to create a non-circular optical fibre [139]. A specially designed extrusion die was used to create the three-lobe form after the filament was directly extruded from plastic pellets. To create a low-cost optical fibre bend direction and rotation sensor, the three-lobe design was developed. The bend direction sensing principle is applied by analyzing how the three-lobe core’s internal light field distribution changes as the plastic filament is bent. The fibre’s circular asymmetries make it possible to determine the direction and degree of rotation. Using a red LED (645 nm) and a charge-coupled device (CCD) positioned in front of the fibre’s end facet, the sensor is queried while it is in transmission. As seen in Figure 9b, the transversal section of the three-lobe fibre was photographed using an optical microscope, processed, and loaded into Lumerical MODE software. The lowest order modes in both the straight and bent fibres’ light fields were calculated using mode simulations. The lowest-order fibre mode’s simulated light field intensity is shown in Figure 9c for the straight fibre [139].

It is stated in [140] that a wide range of applications for POF-based sensors in avionics are feasible. Since the results demonstrate high sensitivity, as well as a high degree of signal repeatability and linearity, the employment of such sensors for strain sensing in avionic structures is quite promising. For monitoring the tip clearance (TC) or blade tip timing (BTT) in aero-engine applications that call for non-contact measurements, compact design, and immunity to electromagnetic interference, fibre-based reflecting optical sensors are also suitable choices. The output of the fibre’s speckle pattern may be utilized to detect periodic changes in the spatial distribution of energy, making a POF an efficient and affordable technique to detect vibrations. As a result of successful temperature and liquid-level discrete monitoring demonstrations, POF-based low-cost and sensitive fibre-optic sensors are now suitable for usage within aircraft tanks. Because the medium is only exposed to light and the fibre’s plastic composition, these sensors offer an appealing replacement for conventional measurement methods. This reduces the possibility of electrical sparks igniting a fire or causing a tank explosion. Additionally, the utilization of energy to remotely power sensor nodes for domestic applications and their potential expansion to avionic situations are also discussed. Power over fibre solutions may be implemented in POF communication lines, producing optical power levels of up to several mW while doing so without affecting data transfer.

To cut down on maintenance time and expense, structural health monitoring is crucial in aircraft transportation. It also enables continuous monitoring of the structures under stress to prevent unsafely high readings. Various suggestions utilizing various types of optical fibres have been reported on by numerous researchers for this purpose. The higher Young’s modulus of POFs, however, makes them stand out as the best choices for the measurement of high strain levels. According to [141,142], it can be helpful to employ a POF elongation sensor to measure the lengthening of an aircraft flap. Figure 9d depicts the setup’s design and basic operation. It works by comparing the phase differences of two modulated light signals. If one of the POFs (POF 1) elongates, the signal that reaches the matching photodetector (Receiver 1) phase shifts [140].

### 4.6. Fibre Bragg Grating (FBG)-Based Sensors

FBG sensors utilize the phenomenon of Bragg reflection to measure various physical variables such as strain, temperature, pressure, and acceleration [143,144]. They are widely used in industries such as aerospace, civil engineering, oil and gas, and structural health monitoring [145]. FBG sensors are based on the principle of Bragg gratings, which are periodic variations in the refractive index of optical fibre [146]. When a narrowband light source, typically a laser, is launched into the fibre, it propagates along the fibre until it encounters the Bragg grating. The Bragg grating acts as a wavelength-specific reflector. It reflects a narrow band of light centred around a particular wavelength known as the Bragg wavelength [147]. The Bragg wavelength is determined by the periodicity of the grating structure and the effective refractive index of the fibre core [148].

However, FBG sensors are not well-suited for high-frequency dynamic measurements due to their limited response time [149]. Rapid changes in strain or temperature can result in incomplete or inaccurate measurements. FBGs are sensitive to environmental conditions and can experience wavelength drift over time. This drift can lead to measurement inaccuracies if not properly compensated for. While FBGs can be multiplexed on a single optical fibre, the process can be complex and require specialized equipment. As the number of sensors increases, managing and demodulating the signals becomes more challenging. Moreover, FBGs are inherently sensitive to both temperature and strain changes [150]. Distinguishing between the two effects can be challenging, particularly in applications with rapidly changing temperature and strain conditions.

When an external parameter, such as strain or temperature, affects the fibre containing the FBG, it modifies the effective refractive index of the fibre. This change in the effective refractive index alters the Bragg wavelength. By measuring the shift in the reflected wavelength, the physical variable can be determined. To measure the shift in the reflected wavelength, a wavelength demodulation system is used. It typically consists of a broadband light source, an OSA, or a wavelength demodulation unit. The system analyses the reflected light spectrum and determines the shift in the Bragg wavelength, which is then correlated to the physical variable being measured. FBG sensors can be used for distributed sensing over long distances, enabling the monitoring of large structures or pipelines. FBG sensors are immune to EMI since they operate based on light signals. FBG sensors are used to monitor the structural integrity of buildings, bridges, dams, and pipelines. They are employed in aircraft wings, fuselages, and turbine engines to measure strain, temperature, and vibration [151]. FBG sensors are used for wellbore monitoring, pipeline integrity, and reservoir monitoring. They are also used in medical devices, such as catheters and endoscopes, for measuring pressure and temperature [152,153]. FBG sensors can detect very small changes in strain, temperature, or other variables. Multiple FBG sensors can be fabricated along a single optical fibre, allowing simultaneous measurement of different variables.

Shi-Zhi Chen et al. [154] developed damage-detection techniques established on long-gauge FBG (LG FBG) for highway bridges. Under these conditions, LG FBG sensors were created as a unique optical sensor to assess the macrostrain response of the structure. Based on this sensor, numerous derivative damage detection methods were presented. Thus, a careful comparison analysis of three typical approaches employing LG FBG was carried out. First, the conceptual frameworks and formats of these methodologies were recast and unified for easier comparability. Then, grounded on verified vehicle–bridge coupling modelling, the performance of these techniques was assessed using a series of parametric experiments, encompassing several damage scenarios, vehicle types, speeds, road roughness, and noise levels. The three approaches’ accuracy and dependability were carefully examined and contrasted.

A. Gizatulin et al. studied the development of fibre vortex mode creation employing chiral periodic structures that contain both chiral optical fibres and chiral (vortex) fibre Bragg gratings (ChFBGs) [155]. An extended theoretical model of the ChFBG was constructed, containing an arbitrary function of apodization, and chirping, which provided a mechanism to compute gratings that produce vortex modes with a particular state for the desired frequency band and reflection coefficient. Additionally, a matrix technique based on the coupled mode theory and scattering matrices mathematical framework was suggested for explaining the ChFBG. It was suggested that ChFBGs be employed as physical field sensors (temperature, tension, etc.) that can be integrated into multi-sensor monitoring systems since they have a special address parameter—the orbital angular momentum of optical light.

The use of addressed fibre Bragg structures (AFBS) with two symmetrical phase shifts in load-sensing wheel hub bearings was described by T. Agliullin et al. in their work [156]. A single AFBS sensor was fitted in the front right wheel hub of an experimental vehicle together with a prototype load-sensing bearing. During the braking test, the experimental setup produced outcomes that were comparable to those of the simulation. To improve the efficiency and safety of active safety systems like automatic braking, adaptive cruise control, and fully automated driving, the proposed system with load-sensing bearings is designed to estimate the loads acting on the wheels. These loads are then used as input parameters for these active safety systems.

Morozov et al. introduced multi-addressed fibre Bragg structures (MAFBS) for microwave-photonic sensor systems [157]. The reflection spectrum of the MAFBS, a unique kind of FBG, contains three or more small notches. The infrared spectrum contains the frequencies of narrow notches, whereas the microwave spectrum contains the variations between them. The address frequencies set refers to all cross-differences between optical frequencies of a single MAFBS. The complex microwave signal, which contains all cross-frequency beatings of all optical frequencies that are incorporated in this optical signal, is collected at the photodetector when the additive optical response from a single MAFBS passed through an optic filter with an oblique amplitude-frequency characteristic is detected. There are enough data in this complicated microwave signal at the photodetector output to ascertain a change in the central frequency of MAFBS [157].

For real-world applications, FBG sensors must often be covered due to their fragility; nevertheless, to prevent temperature-strain cross-sensitivity problems, difficult packaging solutions are needed. For strain-free FBG-based temperature monitoring, a polydimethylsiloxane (PDMS) packaging with a microarray structure that offers dry adhesion inspired by geckos is suggested [158]. In addition to providing protection, the PDMS packaging with a built-in polyamide capillary reduces the mechanical strain that is imparted to the optical fibre, enabling strain-free FBG-based temperature measurement. Additionally, a gecko-inspired dry adhesion based on van der Waals forces is provided by the microarray structure imprinted on one surface of the package. This capability allows the bundled optical fibre sensor to dynamically connect and detach from almost any smooth surface without leaving any remnants in the structure being monitored. The sensor responds to temperature quickly and accurately, and the influence of residual strain is greatly reduced, fitting experimental data. The suggested packed sensor can be utilized in circumstances where using adhesive is either prohibited or not advised.

**Figure 10:**
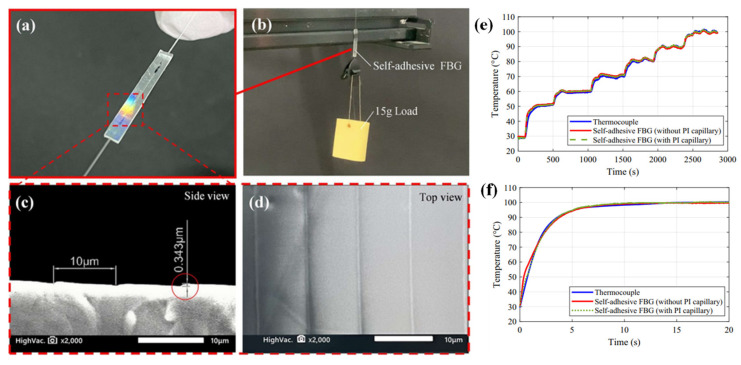
(**a**) Photograph of the manufactured sensing device [158]. (**b**) Self-adhesive FBG with a rubber weight hanging [158]. (**c**) SEM photograph of self-adhesive FBG cross-section [158]. (**d**) SEM photograph of self-adhesive FBG surface [158]. (**e**) Experiment executed using a step-by-step temperature escalation [158]. (**f**) A single 70 °C temperature step [158].

Figure 10a displays an example of the fabricated self-adhesive FBG sensor. On one of the packing sides in the image, light diffraction can be seen to indicate the self-adhesive side. An image of the test in Figure 10b illustrates how the sample’s strong self-adhesion characteristic allowed for the easy hanging of a 15 g rubber weight [158]. Images captured by a scanning electron microscope (SEM) are displayed in Figure 10c,d to confirm the microarray adhesive structure of the fabricated PDMS packaging. With a period of 10 µm and a depth of 0.343 µm, the microarray structure is wedge-shaped, cleanly structured, and equally dispersed, as intended, according to SEM pictures [158]. Figure 10e depicts the temperature development as recorded by the standard thermocouple placed adjacent to the two self-adhesive PDMS-packaged FBG sensors (one with and one without a PI capillary). The thermocouple-provided reference temperature is very closely followed by the temperature evolutions obtained by the two self-adhesive FBG sensors, according to the results. The packed FBG sensors with PI capillary and without PI capillary exhibit root-mean-square errors of 0.44 °C and 0.52 °C, respectively, when comparing temperature data obtained after stabilization. The greatest absolute error, however, was less than 0.7 °C. A temperature step of 70 °C was applied to the aluminium plate to test the two self-adhesive FBG sensors’ ability to respond quickly to temperature changes (both with and without the PI capillary). The temperature increase from 30 °C to 100 °C as recorded by the two samples and the reference thermocouple is shown in Figure 10f [158]. The two packaging types of PDMS-packaged FBG sensors have comparable reaction times to thermocouples, according to the results, verifying the PDMS packaging’s strong thermal responsiveness. The bundled FBG sensors and thermocouple need around 9 s to attain the final temperature, or that amount of time for the measured temperature to change by 98% of the applied thermal variation [158].

## 5. Concluding Statements

Fibre optic sensors play a crucial role in various industries and applications due to their unique advantages and capabilities. They can detect and measure slight variations in parameters such as temperature, pressure, strain, vibration, and chemical composition. They offer high sensitivity, enabling the detection of subtle modifications that may be critical in many applications. These sensors can be used for remote sensing over long distances. Contrasting with traditional electrical sensors, fibre optic sensors are resistant to EMI and can transmit data over hundreds of meters or even kilometres without degradation. This makes them ideal for applications in hazardous or hard-to-reach environments.

Fibre optic sensors can be easily multiplexed, allowing multiple sensors to be connected to a single fibre optic cable. This enables efficient monitoring of multiple parameters simultaneously, reducing installation complexity and costs. These sensors are small and lightweight, making them suitable for applications where space and weight constraints are important factors, such as in aircraft, automobiles, and medical devices.

In this review, six different configurations of fibre optics were discussed for a diverse range of sensing applications. To enhance the sensitivity of any kind of fibre optic-based sensor, the phenomenon of SPR is utilized to identify variations in the refractive index of the surrounding medium. It combines the advantages of fibre optic technology with the sensitivity of SPR. S-shaped fibres are primarily used for strain and curvature measurements. The strain induced by bending affects the transmission characteristics of light in the fibre, allowing for the measurement of parameters such as strain, temperature, pressure, and displacement. S-shaped fibre sensors find applications in structural health monitoring, geotechnical engineering, and industrial processes. U-shaped fibre sensors are created by bending an optical fibre into a U-shaped configuration. Like S-shaped fibre sensors, the bending induces strain in the fibre, enabling the measurement of parameters like strain, temperature, and pressure. U-shaped fibre sensors are utilized in structural health monitoring, civil engineering, and industrial applications. Another widely used type of optical fibre is the D-shaped fibre. D-shaped fibre sensors have a flattened or partially flattened side, resembling the letter “D”. The flattened region enables efficient coupling of light into or out of the fibre. They are commonly used in evanescent field sensing, where the evanescent field interacts with the surrounding medium and is employed in applications such as refractive index sensing, chemical analysis, and biomedical sensing.

The concept of PCFs originated from the study of periodic structures and their influence on the propagation of light. Traditional optical fibres used total internal reflection to guide light within the core, but PCF works on the principle of controlling light through the interaction with a periodic pattern of airholes or voids running along the length of the fibre. By introducing defects or variations in the photonic crystal structure, the fibre can interact with its surroundings and enable sensing capabilities. PhC-based fibre sensors offer high sensitivity and are used for various applications such as temperature sensing, humidity monitoring, strain measurement, and refractive index sensing.

Another interesting type of fibre optic sensor is based on a Bragg grating structure. FBG sensors employ regular variations in the refractive index along the length of an optical fibre to create a wavelength-specific reflection pattern. These gratings act as wavelength-selective filters and are used to measure parameters such as strain, temperature, pressure, and acceleration. A few forms of optical fibre probes, such as FBGs, which are employed in real-time monitoring of deformation in buildings, planes, and ships, are among the few types of fibre-optic probes that have reached the commercialization stage since research on them began 30 years ago. Fibre-optic probes based on the Fabry–Pérot interferometer are currently offered on the market for uses like temperature measurements. Additionally, continuous glucose measurement using Fabry–Pérot fibre probes may soon be used in intensive care units.

POF sensors use plastic fibres instead of traditional glass fibres. The raw materials used to manufacture plastic fibres, such as PMMA or other polymers, are generally less expensive compared to the materials used for glass fibres. This difference in material cost contributes to the overall cost advantage of POFs. POF sensors are often used in applications where electrical insulation, lightweight design, or tight bending radii are required. They find applications in automotive systems, consumer electronics, medical devices, and home automation. It is important to note that while POFs offer cost advantages, they also have certain limitations, such as higher attenuation and lower bandwidth compared to glass fibres. Therefore, the selection of fibre optic technology should consider the specific requirements of the application, including performance, budget, and other factors. Overall, the cost efficiency of POFs makes them an attractive option for applications where their unique characteristics, such as flexibility, ease of handling, and electrical insulation, align with the performance requirements and cost considerations of the application.

## Data Availability

Not applicable.

## Abbreviations

Electromagnetic = EM; Total internal reflection = TIR; Optical coherence tomography = OCT; Surface plasmon resonance = SPR; Localized surface plasmon resonance = LSPR; Addressed fibre Bragg structure = AFBS; Single mode fibre = SMF; Multi-mode fibre = MMF; Plastic optical fibre = POF; Photonic crystal fibre = PCF; polymethyl methacrylate = PMMA; Scanning electron microscope = SEM; Fibre Bragg grating = FBG; Optical time domain reflectometry = OTDR; Optical frequency domain reflectometry = OFDR; Finite element method = FEM; Optical spectrum analyser = OSA.

## References

[1] Butt M.A., Kazanskiy N.L., Khonina S.N., Voronkov G.S., Grakhova E.P., Kutluyarov R.V. (2023). A Review on Photonic Sensing Technologies: Status and Outlook. Biosensors.

[2] Haider F., Aoni R.A., Ahmed R., Mahdiraji G.A., Azman M.F., Adikan F.R.M. (2020). Mode-multiplex plasmonic sensor for multi-analyte detection. Opt. Lett..

[3] Zhdanov A.V., Li L., Yang P., Shkirdova A.O., Tang S., Yashunsky D.V., Ponomarev G.V., Zamilatskov I.A., Papkovsky D.B. (2022). Advanced multi-modal, multi-analyte optochemical sensing platform for cell analysis. Sens. Actuators B Chem..

[4] Swiontek S.E., Pulsifer D.P., Lakhtakia A. (2013). Optical sensing of analytes in aqueous solutions with a multiple surface-plasmon-polariton-wave platform. Sci. Rep..

[5] Butt M.A., Voronkov G.S., Grakhova E.P., Kutluyarov R.V., Kazanskiy N.L., Khonina S.N. (2022). Environmental Monitoring: A Comprehensive Review on Optical Waveguide and Fiber-Based Sensors. Biosensors.

[6] Butt M.A. (2020). Numerical investigation of a small footprint plasmonic Bragg grating structure with a high extinction ratio. Photonics Lett. Pol..

[7] Butt M.A., Khonina S.N., Kazanskiy N.L. (2021). Plasmonics: A Necessity in the Field of Sensing-A Review (Invited). Fiber Integr. Opt..

[8] Butt M.A. (2023). Numerical Assessment of a Metal-Insulator-Metal Waveguide-Based Plasmonic Sensor System for the Recognition of Tuberculosis in Blood Plasma. Micromachines.

[9] Kazanskiy N.L., Khonina S.N., Butt M.A. (2023). Recent Development in Metasurfaces: A Focus on Sensing Applications. Nanomaterials.

[10] Li B., Zhang R., Bi R., Olivo M. (2023). Applications of Optical Fiber in Label-Free Biosensors and Bioimaging: A Review. Biosensors.

[11] Cai J., Liu Y., Shu X. (2023). Long-Period Fiber Grating Sensors for Chemical and Biomedical Applications. Sensors.

[12] Zhu C., Gerald R.E., Huang J., Ossandon M.R., Baker H., Rasooly A. (2022). Micromachined Optical Fiber Sensors for Biomedical Applications. Biomedical Engineering Technologies: Volume 1.

[13] Mulchandani A., Pan S., Chen W. (1999). Fiber-Optic Enzyme Biosensor for Direct Determination of Organophosphate Nerve Agents. Biotechnol. Prog..

[14] Hengoju S., Shvydkiv O., Tovar M., Roth M., Rosenbaum M.A. (2022). Advantages of optical fibers for facile and enhanced detection in droplet microfluidics. Biosens. Bioelectron..

[15] Xie Y., Wang M., Zhong Y., Deng L., Zhang J. (2023). Label-Free Anomaly Detection Using Distributed Optical Fiber Acoustic Sensing. Sensors.

[16] Chen M., He T., Zhao Y., Yang G. (2023). Ultra-short phase-shifted fiber Bragg grating in a microprobe for refractive index sensor with temperature compensation. Opt. Laser Technol..

[17] Paixão T., Nunes A.S., Bierlich J., Kobelke J., Ferreira M.S. (2022). Fabry-Perot Interferometer Based on Suspended Core Fiber for Detection of Gaseous Ethanol. Appl. Sci..

[18] Spammer S.J., Swart P.L., Booysen A. (1996). Interferometric distributed optical-fiber sensor. Appl. Opt..

[19] Muanenda Y., Oton C.J., Di Pasquale F. (2019). Application of Raman and Brillouin Scattering Phenomena in Distributed Optical Fiber Sensing. Front. Phys..

[20] Shatalin S.V., Treschikov V.N., Rogers A.J. (1998). Interferometric optical time-domain reflectometry for distributed optical-fiber sensing. Appl. Opt..

[21] Mermelstein M.D. (1986). All-fiber polarimetric sensor. Appl. Opt..

[22] Jiao L., Zhong N., Zhao X., Ma S., Fu X., Dong D. (2020). Recent advances in fiber-optic evanescent wave sensors for monitoring organic and inorganic pollutants in water. TrAC Trends Anal. Chem..

[23] Liang C., Bai Q., Yan M., Wang Y., Zhang H., Jin B. (2021). A Comprehensive Study of Optical Frequency Domain Reflectometry. IEEE Access.

[24] Singh S., Chaudhary B., Upadhyay A., Sharma D., Ayyanar N., Taya S.A. (2023). A review on various sensing prospects of SPR based photonic crystal fibers. Photonics Nanostructures—Fundam. Appl..

[25] Singh A.K., Mittal S., Das M., Saharia A., Tiwari M. (2023). Optical biosensors: A decade in review. Alex. Eng. J..

[26] Zhang H., Zhou X., Li X., Gong P., Zhang Y., Zhao Y. (2023). Recent Advancements of LSPR Fiber-Optic Biosensing: Combination Methods, Structure, and Prospects. Biosensors.

[27] Wang Z., Zhang W., Liu X., Li M., Lang X., Singh R., Marques C., Zhang B., Kumar S. (2022). Novel Optical Fiber-Based Structures for Plasmonics Sensors. Biosensors.

[28] Du C., Wang Q., Zhao S., Deng X. (2023). Biological sensors based on long period fiber grating. Opt. Laser Technol..

[29] Shi F., Zhang H., Ye Z., Tang X., Qin F., Yan J., Amano H. (2022). Miniature optical fiber curvature sensor via integration with GaN optoelectronics. Commun. Eng..

[30] Liang Y., Wei X., Chu S., Zhang X., Fang Y., Peng W. (2023). Tamm-surface plasmon resonances from nanograting-coupled plasmonic-photonic multilayer structure for an integrated fiber-optic sensing application. J. Phys. Appl. Phys..

[31] Yin Z., Jing X., Bai G., Wu B., Gao Z., Liu C., Wang C., Li Y. (2023). Experimental Study of Dual-Parameter SPR Sensor With Integrated Sensing Channel. IEEE Sens. J..

[32] Khonina S.N., Kazanskiy N.L., Butt M.A., Karpeev S.V. (2022). Optical multiplexing techniques and their marriage for on-chip and optical fiber communication: A review. Opto-Electron. Adv..

[33] Lyu S., Wu Z., Shi X., Wu Q. (2022). Optical Fiber Biosensors for Protein Detection: A Review. Photonics.

[34] Cruz J., Fangueiro R. (2016). Surface Modification of Natural Fibers: A Review. Procedia Eng..

[35] Meunier D., Schruyers J., Gonzales Palla R., Mendoza C., Calberg C., Heinrichs B., Pirard S., Mahy J.G. (2023). Controlled-chemical etching of the cladding in optical fibers for the design of analytical sensors. Opt. Fiber Technol..

[36] Li H., Ni J., Zhao Q., Jiang L. (2023). Surface Modified Optical Fiber Fabry–Perot Cavity Pressure Sensor With Carbon Film. IEEE Sens. J..

[37] Lee B.H., Min E.J., Kim Y.H. (2013). Fiber-based optical coherence tomography for biomedical imaging, sensing, and precision measurements. Opt. Fiber Technol..

[38] Rahmani B., Oguz I., Tegin U., Hsieh J.L., Psaltis D., Moser C. (2022). Learning to Image and Compute with MULTIMODE optical Fibers. Nanophotonics.

[39] Smith A.M. (1978). Polarization and magnetooptic properties of single-mode optical fiber. Appl. Opt..

[40] Savović S., Simović A., Drljača B., Kovačević M.S., Kuzmanović L., Djordjevich A., Aidinis K., Min R. (2023). Power Flow in Multimode Graded-Index Microstructured Polymer Optical Fibers. Polymers.

[41] Highly Sensitive Multi-Core Flat Fiber Surface Plasmon Resonance Refractive Index Sensor. https://opg.optica.org/oe/fulltext.cfm?uri=oe-24-3-2485&id=335915.

[42] Kiroriwal M., Singal P. (2022). Applications of photonic crystal fibers in optical communication. J. Opt. Commun..

[43] Divya J., Selvendran S. (2023). Surface Plasmon Resonance-Based Gold-Coated Hollow-Core Negative Curvature Optical Fiber Sensor. Biosensors.

[44] Shih M., Nelson-Quillin H.D., Garrett K.E., Coyle E.J., Secondo R., Keyser C.K., Mills M.S., Harper E.S. (2023). Maximizing supercontinuum bandwidths in gas-filled hollow-core fibers using artificial neural networks. J. Appl. Phys..

[45] Arroyo E., Tentori D., Garcia A., Valdez R., Armenta M.A., Nava O.J., Machorro R., Olivas A. (2023). Carbon Quantum Dot Optical Properties for potential infiltration into Hollow Core Photonic Crystal Fibers. Part. Part. Syst. Charact..

[46] Gupta B.D., Kant R. (2018). [INVITED] Recent advances in surface plasmon resonance based fiber optic chemical and biosensors utilizing bulk and nanostructures. Opt. Laser Technol..

[47] Tapered Optical Fiber Sensor Based on Localized Surface Plasmon Resonance. https://opg.optica.org/oe/fulltext.cfm?uri=oe-20-19-21693&id=241250.

[48] Liu C., Wang J., Wang F., Su W., Yang L., Lv J., Fu G., Li X., Liu Q., Sun T. (2020). Surface plasmon resonance (SPR) infrared sensor based on D-shape photonic crystal fibers with ITO coatings. Opt. Commun..

[49] Sensitivity Optimization of U-Shaped Fiber Optics Based on the Taguchi Method. https://opg.optica.org/osac/fulltext.cfm?uri=osac-4-7-2024&id=452914.

[50] Johari S.H., Cheak T.Z., Rahim HR A., Jali M.H., Yusof HH M., Johari MA M., Harun S.W. (2022). Formaldehyde Sensing Using Tapered U-Shape Plastic Optical Fiber Coated With Zinc Oxide Nanorods. IEEE Access.

[51] Ren Z.-H., Wang Q., Zhao W.-M., Wang L., Jiang C.-Q., Cong X.-W., Yan X., Zhu A.-S., Qiu F.-M., Chen B.-H. (2022). A High-FOM surface plasmon resonance sensor based on MMF-TUMMF-MMF structure of optical fiber. Opt. Fiber Technol..

[52] Li L., Zhang Y., Zheng W., Li X., Zhao Y. (2022). Optical fiber SPR biosensor based on gold nanoparticle amplification for DNA hybridization detection. Talanta.

[53] Zhang J., Mai X., Hong X., Chen Y., Li X. (2022). Optical fiber SPR biosensor with a solid-phase enzymatic reaction device for glucose detection. Sens. Actuators B Chem..

[54] Kishida K., Imai M., Kawabata J., Guzik A. (2022). Distributed Optical Fiber Sensors for Monitoring of Civil Engineering Structures. Sensors.

[55] Ashry I., Mao Y., Wang B., Hveding F., Bukhamsin A.Y., Ng T.K., Ooi B.S. (2022). A Review of Distributed Fiber–Optic Sensing in the Oil and Gas Industry. J. Light. Technol..

[56] Liu T., Wei Y., Song G., Hu B., Li L., Jin G., Wang J. (2018). Fibre Optic Sensors for Coal Mine Hazard Detection. Measurement.

[57] Schenato L. (2017). A Review of Distributed Fibre Optic Sensors for Geo-Hydrological Applications. Appl. Sci..

[58] Bado M.F., Casas J.R. (2021). A Review of Recent Distributed Optical Fiber Sensors Applications for Civil Engineering Structural Health Monitoring. Sensors.

[59] Distributed Fiber Optic Sensor Market Share Report, 2030. https://www.grandviewresearch.com/industry-analysis/distributed-fiber-optic-sensor-sensing-dfos-market.

[60] Nedoma J., Fajkus M., Martinek R., Vanus J., Kepak S., Kahankova R., Jaros R., Cvejn D., Prauzek M. (2018). Analysis of the use of fiber-optic sensors in the road traffic. IFAC-Pap..

[61] García I., Zubia J., Durana G., Aldabaldetreku G., Illarramendi M.A., Villatoro J. (2015). Optical Fiber Sensors for Aircraft Structural Health Monitoring. Sensors.

[62] Lorincz J., Klarin Z., Begusic D. (2023). Advances in Improving Energy Efficiency of Fiber–Wireless Access Networks: A Comprehensive Overview. Sensors.

[63] Bednarz B., Popielski P., Sieńko R., Howiacki T., Bednarski Ł. (2021). Distributed Fibre Optic Sensing (DFOS) for Deformation Assessment of Composite Collectors and Pipelines. Sensors.

[64] Liu Z., Wang L., Meng Y., He T., He S., Yang Y., Xiao Q. (2022). All-Fiber High-Speed Image Detection Enabled by Deep Learning. Nat. Commun..

[65] Mittal N., Shah M., John J. (2016). A Low Cost Short Haul Plastic Optical Fiber Link for Home Networking Applications. Proceedings of the 2016 IEEE International Conference on Recent Trends in Electronics, Information & Communication Technology, Bangalore, India, 20–21 May 2016.

[66] Xu J., Li T., Li Y., Zhang C., Cheng L., Liu L., Miao C. (2023). Fabrication and application of a grooved optical fiber respiratory sensor based on geometric parameter optimization by optical simulation. Opt. Laser Technol..

[67] Huang L., Liu S., Zheng B., Zhao Y., Dang L., Gao L., Huang W., Yin G., Zhu T. (2023). Narrowband all-fiber acousto-optic tunable add-drop filter based on dispersion-compensating fiber. Opt. Laser Technol..

[68] Wieduwilt T., Förster R., Nissen M., Kobelke J., Schmidt M.A. (2023). Characterization of diffusing sub-10 nm nano-objects using single anti-resonant element optical fibers. Nat. Commun..

[69] Shao M., Cao Z., Gao H., Yu D., Qiao X. (2023). Optical Fiber Ultrasonic Sensor Based on Partial Filling PDMS in Hollow-Core Fiber. Opt. Laser Technol..

[70] Home. https://fiso.com/en/.

[71] Luna Innovations|Fiber Optic Sensing and Measurement Systems. https://lunainc.com/.

[72] Micron Optics, Inc. SENSORS. http://micronoptics.ru/sensors_products.html.

[73] OSENSA Innovations Corp OSENSA Innovations. https://www.osensa.com/.

[74] Opsens Fiber Optic Sensor Company for FFR and Oil & Gas. https://opsens.com/.

[75] Neubrex Co., Ltd. Home. https://www.neubrex.com/.

[76] Advanced Fiber Optic DTS & Monitoring Systems—Sensornet. https://www.sensornet.co.uk/.

[77] Uniyal A., Srivastava G., Pal A., Taya S., Muduli A. (2023). Recent Advances in Optical Biosensors for Sensing Applications: A Review. Plasmonics.

[78] De A., Kalita D. (2023). Bio-Fabricated Gold and Silver Nanoparticle Based Plasmonic Sensors for Detection of Environmental Pollutants: An Overview. Crit. Rev. Anal. Chem..

[79] Chen Y., Li H., Li B., Wan L., Wang L. (2022). Fault diagnosis of fiber optic current sensor induced by light source based on support vector machines. Proceedings of the International Conference on Electronic Information Technology (EIT 2022), Chengdu, China, 18–20 March 2022.

[80] Vahdati N., Shiryayev O., Parapurath S.M., Yap F.F., Butt H. (2022). Cost-Effective Corrosion Detection Sensor for Above-Ground Oil and Gas Flowlines. Sensors.

[81] Liu W., Liu Z., Zhang Y., Li S., Zhang Y., Yang X., Zhang J., Yuan L. (2022). Specialty optical fibers and 2D materials for sensitivity enhancement of fiber optic SPR sensors: A review. Opt. Laser Technol..

[82] Zhu C., Zhuang Y., Liu B., Huang J. (2022). Review of Fiber Optic Displacement Sensors. IEEE Trans. Instrum. Meas..

[83] Losch M.S., Kardux F., Dankelman J., Hendriks B.H.W. (2022). Steering light in fiber-optic medical devices: A patent review. Expert Rev. Med. Devices.

[84] Applications of Fiber-Optic Biochemical Sensor in Microfluidic Chips: A Review—ScienceDirect. https://www.sciencedirect.com/science/article/pii/S0956566320304413?via%3Dihub.

[85] Mittal S., Sharma T., Tiwari M. (2021). Surface plasmon resonance based photonic crystal fiber biosensors: A review. Mater. Today Proc..

[86] Pendão C., Silva I. (2022). Optical Fiber Sensors and Sensing Networks: Overview of the Main Principles and Applications. Sensors.

[87] Dejdar P., Mokry O., Cizek M., Rajmic P., Munster P., Schimmel J., Pravdova L., Horvath T., Cip O. (2023). Characterization of sensitivity of optical fiber cables to acoustic vibrations. Sci. Rep..

[88] Huang Q., Shi H., Huang C., Sun J. (2023). Improvement of response speed and precision of distributed Brillouin optical fiber sensors using neural networks. Opt. Laser Technol..

[89] Tian Y., Dang H., Liu W., Cui J., Li Y., Tan J. (2023). Structure shape measurement method based on an optical fiber shape sensor. Meas. Sci. Technol..

[90] Singh M., Raghuwanshi S.K., Prakash O., Saini P.K. (2020). High-Resolution Fiber Optic Sensor based on Coated Linearly Chirped Bragg Grating. Optik.

[91] Zheng Y., Shum P.P., Luo Y., Zhang Y., Ni W., Wang G., Wu Z., Dinh X.Q., Auguste J.-L., Humbert G. (2020). High-resolution, large-dynamic-range multimode interferometer sensor based on a suspended-core microstructured optical fiber. Opt. Lett..

[92] Alabbas S.H., Ashworth D.C., Bezzaa B., Momin S.A., Narayanaswamy R. (1995). Factors affectig the response time of an optical-fibre reflectance pH sensor. Sens. Actuators Phys..

[93] Dooly G., Manap H., O’Keeffe S., Lewis E. (2011). Highly Selective Optical Fibre Ammonia Sensor for use in Agriculture. Procedia Eng..

[94] Xiong Y., Shepherd S., Tibbs J., Bacon A., Liu W., Akin L.D., Ayupova T., Bhaskar S., Cunningham B.T. (2023). Photonic Crystal Enhanced Fluorescence: A Review on Design Strategies and Applications. Micromachines.

[95] Kuiri B., Dutta B., Sarkar N., Atta R., Mallick K., Sharma M.D., Patra A.S. (2023). Development of ring-core photonic crystal fiber based on LiNbO3 supporting higher-order OAM modes. Opt. Quantum Electron..

[96] Wang X., Li S., Cheng T., Li J. (2022). Overview of photonic devices based on functional material-integrated photonic crystal fibers. J. Phys. Appl. Phys..

[97] Gao Y., Yan X., Chen X., Li B., Cheng T. (2023). A Refractive Index Sensor Based on Four-Wave Mixing in D-Shaped Tellurite Photonic Crystal Fiber. Photonic Sens..

[98] Wang H., Wu M., Zheng S., Xie T., Dai W., Fu H. (2023). Surface plasmon resonance sensor based on seven-core photonic crystal fiber for refractive index and temperature measurement. Opt. Laser Technol..

[99] Hou Y.C., Li Y.F., Xie X.F., Kou Z.L., Lu Y., Chen S.Y., Lu Z. (2021). Ion-Doped Photonic Crystal Fiber Lasers. Front. Chem..

[100] Fan Z., Chu S., Zhang X., Meng J., Fan Y., Zhang Y. (2023). Two Kinds of Liquid Crystal Filled PCFs Temperature and RI Sensors Based on SPR. IEEE Sens. J..

[101] Zhang Y., Zhou L., Qiao D., Liu M., Yang H., Meng C., Miao T., Xue J., Yao Y. (2022). Progress on Optical Fiber Biochemical Sensors Based on Graphene. Micromachines.

[102] Liu X., Yin B., Li H., Wang M., Yan R., Li Y., Zong C., Wu S. (2023). Highly sensitive sensor for simultaneous underwater measurement of salinity and temperature based on highly birefringent asymmetric photonic crystal fiber. Results Opt..

[103] Plasmonic Sensing Devices|4|Plasmonics-Based Optical Sensors and D. https://www.taylorfrancis.com/chapters/edit/10.1201/9781003438304-4/plasmonic-sensing-devices-muhammad-ali-butt-svetlana-nikolaevna-khonina-nikolay-lvovich-kazanskiy?context=ubx&refId=27c1332f-8c91-426d-8ff8-b784154efdb5.

[104] Chao C.-T.C., Chen S.-H., Huang H.J., Kooh M.R.R., Lim C.M., Thotagamuge R., Mahadi A.H., Chau Y.-F.C. (2023). Improving Temperature-Sensing Performance of Photonic Crystal Fiber via External Metal-Coated Trapezoidal-Shaped Surface. Crystals.

[105] Abdelghaffar M., Gamal Y., El-Khoribi R.A., Soliman W., Badr Y., Hameed M.F.O., Obayya S.S.A. (2023). Highly sensitive V-shaped SPR PCF biosensor for cancer detection. Opt. Quantum Electron..

[106] Maidi A.M., Kalam M.A., Begum F. (2023). Photonic crystal fibre for blood components sensing. Sens. Bio-Sens. Res..

[107] Alghamdi T.A., Adwan S., Arof H., Harun S.W. (2023). Black Phosphorus Coated D-Shape Fiber as a Mode-Locker for Picosecond Soliton Pulse Generation. Crystals.

[108] Osamah S., Alwahib A.A., Fakhri M.A., Gopinath S.C.B. (2023). Study of single and symmetrical D-shaped optical fiber sensor based on gold nanorods. J. Opt..

[109] Ren Q., Liu F., Miao Y., Zhang K. (2022). Dual-band D-shaped SPR fiber sensor based on birefringence analysis. Opt. Commun..

[110] Osamah S., Alwahib A.A., Fakhri M.A. (2022). D-shape optical fibers based on gold nanoparticles for a different sensors: A review. AIP Conf. Proc..

[111] Zhang Q., Li W., Ren Q., Zheng J., Xie Q., Wang X. (2022). A D-type dual side-polished, highly sensitive, plasma refractive index sensor based on photonic crystal fiber. Front. Phys..

[112] Pan H., Cui N., Pan F., Zhang A., Cao C., Sui P. (2023). High-sensitivity surface plasmon resonance refractive index sensor with high resolution based on D-shaped photonic crystal fiber. J. Opt..

[113] Liang H., Shen T., Feng Y., Liu H., Han W. (2021). A D-Shaped Photonic Crystal Fiber Refractive Index Sensor Coated with Graphene and Zinc Oxide. Sensors.

[114] Chen C.-H., Tsao T.-C., Tang J.-L., Wu W.-T. (2010). A Multi-D-Shaped Optical Fiber for Refractive Index Sensing. Sensors.

[115] Yang R., Yu Y.-S., Xue Y., Chen C., Chen Q.-D., Sun H.-B. (2011). Single S-tapered fiber Mach–Zehnder interferometers. Opt. Lett..

[116] Zhao W., Xiong M., Chen M., Cheng Y., Deng S., Liu H., Teng C., Yang H., Deng H., Yuan L. (2022). Simulation study of a temperature-calibrated double-sided polished optical fiber SPR refractive index sensor. Appl. Opt..

[117] Chow C.W.K., Rameezdeen R., Chen G.Y., Xu H., Rahman M.M., Ma X., Zhuge Y., Gorjian N., Gao J. (2023). Real-Time Humidity Monitoring Using Distributed Optical Sensor for Water Asset Condition Assessment. Water Conserv. Sci. Eng..

[118] Ernewein B., Woods J.E. (2023). Distributed fiber optic strain sensing in axially loaded glued-in steel rods in glued-laminated timber. Constr. Build. Mater..

[119] Lei X.Q., Peng B.J., Chen D.R., Shi Q.G., Ma X.W. (2016). An All-Fiber Magnetic Field Sensor Based on Dual-S-Shaped Optic Fiber Integrated With Magnetic Fluid. IEEE Sens. J..

[120] Chauhan S.K., Tharion J., Punjabi N., Sharma D.K., Mukherji S. (2014). A comparison of S-shaped and U-shaped optical fiber sensors. Proceedings of the Advanced Photonics (2014).

[121] Chauhan S., Punjabi N., Sharma D., Mukherji S. (2016). Evanescent Wave Absorption Based S-shaped Fiber-optic Biosensor for Immunosensing Applications. Procedia Eng..

[122] Dass S., Kachhap S., Jha R. (2019). S-shaped Microfiber Based Diaphragm Supported Optical Microphone. J. Phys. Photonics.

[123] Yan H., Li P., Zhang H., Shen X., Wang Y. (2017). A Micro S-shaped Optical Fiber Temperature Sensor Based on Dislocation Fiber Splice. Photonic Sens..

[124] Wen H.-Y., Hsu H.-C., Tsai Y.-T., Feng W.-K., Lin C.-L., Chiang C.-C. (2021). U-Shaped Optical Fiber Probes Coated with Electrically Doped GQDs for Humidity Measurements. Polymers.

[125] Wang S., Zhang D., Xu Y., Sun S., Sun X. (2020). Refractive Index Sensor Based on Double Side-Polished U-Shaped Plastic Optical Fiber. Sensors.

[126] Song H., Zhang H., Sun Z., Ren Z., Yang X., Wang Q. (2019). Triangular silver nanoparticle U-bent fiber sensor based on localized surface plasmon resonance. AIP Adv..

[127] Gupta B.D., Dodeja H., Tomar A.K. (1996). Fibre-optic evanescent field absorption sensor based on a U-shaped probe. Opt. Quantum Electron..

[128] Chou Y.-L., Wen H.-Y., Weng Y.-Q., Liu Y.-C., Wu C.-W., Hsu H.-C., Chiang C.-C. (2022). A U-Shaped Optical Fiber Temperature Sensor Coated with Electrospinning Polyvinyl Alcohol Nanofibers: Simulation and Experiment. Polymers.

[129] Biswas R. (2022). Catheter like U-shaped fiber as a probe for oral cancer. Biosens. Bioelectron. X.

[130] Okazaki T., Kamio H., Yoshioka M., Ueda A., Kuramitz H., Watanabe T. (2022). U-shaped plastic optical fiber sensor for scale deposition in hot spring water. Anal. Sci..

[131] Goya K., Sasanuma H., Ishida G., Uehara H., Tokita S. (2023). Fusion splicing of plastic optical fibers using a mid-IR fiber laser. Appl. Phys. Express.

[132] Zubia J., Arrue J. (2021). Plastic Optical Fibers: An Introduction to Their Technological Processes and Applications. Opt. Fiber Technol..

[133] Amoiropoulos K., Kioselaki G., Kourkoumelis N., Ikiades A. (2020). Shaping Beam Profiles Using Plastic Optical Fiber Tapers with Application to Ice Sensors. Sensors.

[134] Zhao H., Wang F., Cheng P. (2023). A plastic optic fiber sensor with temperature compensation for refractive index measurement. Opt. Fiber Technol..

[135] Prabowo J., Seo H., Park J. Plastic Optical Fiber AC Voltage Sensor. Proceedings of the 2020 International Conference Laser Optics (ICLO).

[136] Pal T., Aditya S., Mathai T., Mukherji S. (2023). Polyaniline coated plastic optic fiber biosensor for detection of aflatoxin B1 in nut, cereals, beverages, and body fluids. Sens. Actuators B Chem..

[137] Zhao H., Wang F., Wang Z., Chen L. (2023). Refractive index sensor based on a gradually hot-pressed flatted plastic optical fiber. Opt. Commun..

[138] Liu L., Deng S., Zheng J., Yuan L., Deng H., Teng C. (2021). An Enhanced Plastic Optical Fiber-Based Surface Plasmon Resonance Sensor with a Double-Sided Polished Structure. Sensors.

[139] Sartiano D., Geernaert T., Torres Roca E., Sales S. (2020). Bend-Direction and Rotation Plastic Optical Fiber Sensor. Sensors.

[140] Lallana P.C., Aldabaldetreku G., López A., Montero D.S., Durana G., Mateo J., Losada M.Á., Zubia J., Vázquez C. (2021). Sensing Applications in Aircrafts Using Polymer Optical Fibres. Sensors.

[141] Durana G., Kirchhof M., Luber M., de Ocariz I.S., Poisel H., Zubia J., Vazquez C. (2009). Use of a Novel Fiber Optical Strain Sensor for Monitoring the Vertical Deflection of an Aircraft Flap. IEEE Sens. J..

[142] Gomez J., Zubia J., Aranguren G., Arrue J., Poisel H., Saez I. (2009). Comparing polymer optical fiber, fiber Bragg grating, and traditional strain gauge for aircraft structural health monitoring. Appl. Opt..

[143] Xu S., Li X., Wang T., Wang X., Liu H. (2023). Fiber Bragg grating pressure sensors: A review. Opt. Eng..

[144] Mishra M., Sahu P.K. (2023). Fiber Bragg Gratings in Healthcare Applications: A Review. IETE Tech. Rev..

[145] Zhou Z., Xu Y., Qiao C., Liu L., Jia Y. (2021). A novel low-cost gas sensor for CO_2_ detection using polymer-coated fiber Bragg grating. Sens. Actuators B Chem..

[146] Braunfelds J., Haritonovs E., Senkans U., Kurbatska I., Murans I., Porins J., Spolitis S. (2022). Designing of Fiber Bragg Gratings for Long-Distance Optical Fiber Sensing Networks. Model. Simul. Eng..

[147] Butt M.A., Kazanskiy N.L., Khonina S.N. (2022). Advances in Waveguide Bragg Grating Structures, Platforms, and Applications: An Up-to-Date Appraisal. Biosensors.

[148] Bonopera M. (2022). Fiber-Bragg-Grating-Based Displacement Sensors: Review of Recent Advances. Materials.

[149] Hunze A., Badcock R.A., Fisser M. (2018). Response Time of a Fiber Bragg Grating Based Hydrogen Sensor for Transformer Monitoring. Proceedings.

[150] Zhao M., Wang S., Luo B., Zhong N., Cao X. Theoretical Study on the Cross Sensitivity of Fiber Bragg Grating Sensor Affected by Temperature and Transverse Pressure. Proceedings of the 2010 Symposium on Photonics and Optoelectronics.

[151] Akinyemi T.O., Omisore O.M., Duan W., Lu G., Al-Handerish Y., Han S., Wang L. (2021). Fiber Bragg Grating-Based Force Sensing in Robot-Assisted Cardiac Interventions: A Review. IEEE Sens. J..

[152] Sun X., Zeng L., Hu Y., Duan J. (2022). Fabrication and Sensing Application of Phase Shifted Bragg Grating Sensors. Materials.

[153] Xu Z., Shu X., Fu H. (2019). Fiber Bragg grating sensor interrogation system based on an optoelectronic oscillator loop. Opt. Express.

[154] Chen S.-Z., Feng D.-C., Han W.-S. (2020). Comparative Study of Damage Detection Methods Based on Long-Gauge FBG for Highway Bridges. Sensors.

[155] Gizatulin A., Meshkov I., Vinogradova I., Bagmanov V., Grakhova E., Sultanov A. (2020). Generation of Vortex Optical Beams Based on Chiral Fiber-Optic Periodic Structures. Sensors.

[156] Agliullin T., Gubaidullin R., Sakhabutdinov A., Morozov O., Kuznetsov A., Ivanov V. (2020). Addressed Fiber Bragg Structures in Load-Sensing Wheel Hub Bearings. Sensors.

[157] Morozov O., Sakhabutdinov A., Anfinogentov V., Misbakhov R., Kuznetsov A., Agliullin T. (2020). Multi-Addressed Fiber Bragg Structures for Microwave-Photonic Sensor Systems. Sensors.

[158] Liu S., Zhu P., Xie F., Soto M.A. (2023). Gecko-inspired self-adhesive packaging for strain-free temperature sensing based on optical fibre Bragg gratings. Sci. Rep..

